# The Knowledge, Attitudes, and Practices of Healthy Eating Questionnaire: a pilot validation study in Chinese families

**DOI:** 10.3389/fpubh.2024.1355638

**Published:** 2024-07-17

**Authors:** Kiki S. N. Liu, Julie Y. Chen, Kai-Sing Sun, Joyce P. Y. Tsang, Patrick Ip, Carlos K. H. Wong, Cindy L. K. Lam

**Affiliations:** ^1^Department of Family Medicine and Primary Care, School of Clinical Medicine, Li Ka Shing Faculty of Medicine, The University of Hong Kong, Hong Kong, Hong Kong SAR, China; ^2^Department of Family Medicine, The University of Hong Kong Shenzhen Hospital, Shenzhen, China; ^3^JC School of Public Health and Primary Care, The Chinese University of Hong Kong, Hong Kong, Hong Kong SAR, China; ^4^School of Health Sciences, Saint Francis University, Hong Kong, Hong Kong SAR, China; ^5^Department of Paediatrics and Adolescent Medicine, School of Clinical Medicine, Li Ka Shing Faculty of Medicine, The University of Hong Kong, Hong Kong, Hong Kong SAR, China; ^6^Department of Pharmacology and Pharmacy, Li Ka Shing Faculty of Medicine, The University of Hong Kong, Hong Kong, Hong Kong SAR, China; ^7^Laboratory of Data Discovery for Health Limited (D24H), Hong Kong, Hong Kong SAR, China; ^8^Department of Infectious Disease Epidemiology and Dynamics, Faculty of Epidemiology and Population Health, London School of Hygiene and Tropical Medicine, London, United Kingdom

**Keywords:** Chinese, healthy eating, KAP, questionnaire, validation

## Abstract

**Introduction:**

Identifying the knowledge, attitudes, and practices (KAP) gaps of healthy eating can inform the design of effective interventions. This study aimed to test the validity and psychometric properties of a KAP of Healthy Eating Questionnaire (KAP-HEQ) tailored to the Chinese culture.

**Methods:**

The dimensions and potential items of each KAP scale were identified from published KAP and health literacy questionnaires, which were supplemented by the findings of a previous qualitative healthy eating study. Content validity of the KAP-HEQ was evaluated by eight experts and eight Chinese parent–adolescent dyads in Hong Kong through content validity ratio (CVR), content validity index (CVI), and qualitative feedback. The feasibility, construct validity, reliability, and sensitivity of the KAP-HEQ were evaluated in this pilot study among 60 adolescent–parent dyads (120 persons) through an online survey. The first 30 dyads who completed the KAP-HEQ were invited to repeat the KAP-HEQ 2 weeks later to assess the test–retest reliability.

**Results:**

The final 44-item KAP-HEQ was completed in 10–15 min by both adolescents and their adult parents. The CVR ranged from −0.38 to 1, and the CVI ranged from 0.56 to 1. Over 80% of the items achieved convergent validity (a significantly positive correlation with its hypothesized scale) and discriminant validity (a higher correlation with its hypothesized scale than with the other two scales). Cronbach’s alpha for the internal consistency of the Overall, Attitude, and Practice scales was >0.7, while that of the Knowledge scale was 0.54. The intraclass correlation coefficient (ICC) on test–retest reliability of the Overall and individual scales were all >0.75 except that of the Knowledge scale (ICC = 0.58). The significant differences in KAP scale scores with small to large effect sizes were found between known groups as hypothesized, except the Attitude score between groups by household income, which supported the sensitivity of the KAP-HEQ.

**Conclusion:**

The KAP-HEQ has shown good validity, reliability, and sensitivity among Chinese adolescents and adults, which can be applied to evaluate KAP status and gaps to inform the design and assess the effectiveness of healthy eating interventions.

## 1 Introduction

An unhealthy diet predisposes to obesity and non-communicable diseases (NCDs) such as cardiovascular diseases, cancers, and diabetes mellitus (1, 2). Previous studies have shown that diet-related NCDs accounted for more than one-fifth of global deaths among adults and about half of the registered deaths in Hong Kong (3, 4). The low adherence to the dietary guidelines in the local population highlights the need for more effective interventions to promote healthy eating in the local setting (5).

Identifying the knowledge, attitudes, and practices (KAP) gaps of healthy eating is useful to guide the design of nutrition education and interventions (6). The Food and Agriculture Organization of the United Nations has established guidelines for assessing nutrition-related knowledge, attitudes, and practices (7). They specify the key KAP indicators, such as knowledge of dietary recommendations, attitudes toward susceptibility to health consequences from an unhealthy diet, and practices of specific food intake. Several modules in these guidelines contain items that relate to malnutrition and food safety, which are less relevant to developed regions like Hong Kong. We found a variety of questionnaires on knowledge, attitudes, and/or practices of healthy eating in the literature with wide variation in the item content specific to the respective research questions. Some targeted specific food groups, such as fruit and vegetables, whole grains, and salt (8–10), and some were designed to assess the effect of specific interventions such as nutrition education and life skill training (11–13). The questionnaire length varied widely from 10 (14) to 97 items (13). There is also uncertainty about cross-study application, as most questionnaires were administered exclusively within the context of a particular research study.

The utility of an instrument relies on the evaluation of its psychometrics. The traditional psychometrics of an instrument include content validity, construct validity, reliability, and sensitivity (15). Content validity refers to the extent to which the items of the instrument represent the content that is intended to be measured based on the importance, relevance, clarity, and comprehensiveness of the items (16). It is commonly evaluated by experts in the field of study, supplemented by feedback from lay persons of the target population. Content validity of each item can be assessed quantitatively by the content validity ratio (CVR) and content validity index (CVI), and qualitatively by cognitive debriefing (16, 17).

Construct validity refers to whether the items represent the theoretical scale structure. Internal construct validity is commonly assessed by item-scale correlation (18, 19). Known-group comparison assesses external construct validity in that groups hypothesized to have better outcomes will have higher scores (19). It can also evaluate the sensitivity of an instrument to detect a difference between groups. Another common method to assess construct validity is the score correlation between the new instrument and another instrument that measures a similar or different construct (20).

Reliability refers to the ability of the instrument to give consistent and reproducible results. It is commonly assessed by the internal consistency of items in the same scale and test–retest reliability (18).

Among the KAP questionnaires on healthy eating reported in the literature, a few have been shown to be valid and reliable in Asian populations (10, 13, 21). Two of these questionnaires measure the KAP of a healthy lifestyle, including healthy eating and physical activity, among adolescents in Malaysia and India, respectively (13, 21). However, they each comprise over 80 items that may be too burdensome for adolescents to complete (22). The cultural differences in eating habits also limit their applicability in the Chinese population. There is an existing KAP questionnaire focusing on salt intake in Chinese older adults in Hong Kong (10), but we could not find any other questionnaire that assesses healthy eating among the Chinese general population. This calls for the development of a new KAP of healthy eating questionnaire tailored to the Chinese culture.

This study aimed to test the validity and psychometric properties of a new KAP of Healthy Eating Questionnaire (KAP-HEQ) in the Chinese population in Hong Kong. The instrument was designed to be generic and applicable to adolescents and adults.

## 2 Methods

This study consisted of three parts: (1) KAP-HEQ development, (2) evaluation of content validity, and (3) pilot test on psychometrics. Ethics approval was obtained from the Institutional Review Board of the University of Hong Kong/Hospital Authority Hong Kong West Cluster (UW 22-289) for the study design and analytical plan.

### 2.1 KAP-HEQ development

The dimensions and potential items on KAP of healthy eating were identified from published KAP and health literacy questionnaires specific to Asian populations (10, 21, 23, 24) and supplemented by the findings from our previous qualitative studies (25, 26).

The Knowledge scale included the following three dimensions: (1) dietary recommendations, (2) health outcomes (or diet–disease relationship), and (3) healthy food choices. The Attitudes scale included the following three dimensions: (1) outcome expectation that covered perceived susceptibility and benefits, (2) preference, and (3) self-efficacy toward healthy eating. The Practices of healthy eating scale were categorized into the following three dimensions: (1) meal pattern, (2) consumption of healthy food, and (3) eating unhealthy food. Items were selected from the item pool based on their relevance and clarity. The items on food examples and eating habits were tailored to the Chinese culture and local context, such as pastries sold in local bakery stores, and menu choices at Hong Kong-style cafés. While items on fruit and vegetables (FV) intake, as well as salt and sugar intake, were specific, fat intake was difficult to quantify. Therefore, it was assessed using proxy items such as eating out or getting takeaway food, consumption of high-fat snacks, and choosing food with gravy.

Indicators of each dimension were determined and constructed into question items of the KAP-HEQ. A team of three co-investigators [a nutritionist (KL), an expert in patient-reported outcome (PRO) measures (CL), and a dietitian (JT)] reviewed the wording and layout of the items to ensure clarity. The first draft of KAP-HEQ consisted of 48 items.

### 2.2 Evaluation of content validity

The content validity of the 48-item questionnaires was evaluated by both the experts and lay persons from the target population. The recommended number of experts in a panel review is at least five to minimize the chance of agreement (17). We invited a panel of eight expert professionals to evaluate the content validity of the instrument. The panel included two experts in PRO measures (CL and EC), a dietitian (JT), a primary care doctor (ET), a statistician (CW), a pediatrician (PI), and two co-investigators of the qualitative study (JC and KS). Each panelist was sent the draft questionnaires with the definition of each construct and dimension together with an evaluation guide (see Supplementary Data Sheets 1).

Content validity was evaluated by both quantitative rating and qualitative feedback. The first measure was the content validity ratio (CVR), which was based on the importance rating (1 “Not necessary,” 2 “Useful but not essential,” and 3 “Essential”). The CVR was calculated by the ratio of (N_e_ − N/2)/(N/2), where N_e_ is the number of panelists rated “Essential” and N is the total number of panelists, i.e., *N* = 8. According to the Lawshe Table, items with a CVR of 0.75 or above are considered acceptable with 8 panelists, i.e., at least 7 panelists rating “Essential” is required (17). The second method was the content validity index (CVI) on item relevance and clarity, using a 4-point rating from 1 “Not relevant” (or “Not clear”) to 4 “Very relevant” (or “Very clear”) (16). The CVI of each item was calculated by dividing the number of panelists rating 3 or 4 by the total number of panelists. A CVI of ≥0.78 supports the appropriateness (27). The third method was an overall evaluation of the comprehensiveness of the items of each dimension with an open-ended question on “suggestion on addition or deletion of items” at the end of each dimension to collect qualitative feedback from the panelists.

We referenced the COSMIN Study Design checklist for patient-reported outcome measurement instruments (28) for content evaluation by lay persons from the target population. We evaluated the item importance in addition to relevance, clarity (i.e., comprehensibility in the COSMIN), and comprehensiveness of the questionnaires. Due to the limitation of time and resources in this pilot study, we could only recruit a total of 8 parent–adolescent dyads (*N* = 16) from families who participated in an earlier qualitative study on adolescent KAP of healthy eating and the associated family factors (25, 26). There is no standard guideline on the analytical approach (19), and we used the same methods as for expert-reviewed content evaluation. We set standards that were lower than that for experts in that the content would be considered valid if at least half of the lay persons rated the item “Essential” (CVR ≥ 0) and the CVI on relevance or clarity was at least 0.75. Two researchers were involved in analyzing the rating scores. The research team reconciliated and revised the items of the questionnaires based on the results on CVR, CVI, and qualitative feedback from the expert panel and lay persons. Four items that had low content validity ratings were excluded to form the final draft with 44 items (see Supplementary Data Sheets 2).

### 2.3 Pilot test on psychometrics

#### 2.3.1 Subjects and sample size

The sampling population was the families of an existing cohort study to evaluate the effectiveness of a health empowerment program in low-income families (29). There were 390 families in the cohort study with adolescents aged 12–19 years, whose parents were the primary caregivers as of 1 September 2022. A total of 21 families who participated in our previous qualitative study (25, 26) were excluded. A sample size of 100 is generally considered sufficient for a pilot test on the psychometrics of a questionnaire using item analysis (18), and we targeted 120 persons of 60 parent–adolescent dyads.

We field-tested the survey administration with 40 cohort families in September 2022 by sending them a WhatsApp message with a brief introduction to the study aim, eligibility criteria and procedures, and a link for online registration. All registrants were invited to complete the online questionnaire within a week with reminder messages sent on the 5th and last days. We found the online survey process satisfactory with 11 families registered (33.3% of families with WhatsApp contacts), and 10 families completed the survey (90.9%). The online survey invitation was sent to all remaining eligible cohort families via WhatsApp messages from September to October 2022, and they were encouraged to refer their friends to participate. Among the 302 cohort families with WhatsApp contacts, 56 families registered (18.5%) and 49 completed the questionnaires (87.5%). We received referrals from an additional 30 families, and 25 of them completed the survey (83.3%). In total, there were 12 families with incomplete surveys: 4 were completed by the parents only, and the remaining 8 had no response from either parents or adolescents. The data of the first 60 parent–adolescent dyads who completed the survey were included in the pilot test.

#### 2.3.2 Data collection

The survey was administered online in October 2022 using Qualtrics, a survey tool provided by the University of Hong Kong that stored the data in a secure server of the University. Each member of the participating parent–adolescent dyads received a link to the parent- or adolescent-specific survey questionnaire, and a reference code unique to each family. The first page of the survey link contained detailed study information and a consent form that must be completed before proceeding to the questionnaire survey. The parent had to provide the consent on behalf of self and his/her child, and the adolescent had to provide the consent for self. Each participant opted for either “Agree” to proceed to the survey questions or “Disagree” to leave the survey.

The first 30 families who completed the questionnaire were invited to repeat the same questionnaires 2 weeks later to assess test–retest reliability.

#### 2.3.3 Survey instruments

Both adolescents and parents answered the same 44-item KAP-HEQ, along with 2 and 9 items on sociodemographic information, respectively. Parents had 35 additional questions related to family factors to be examined in another study.

#### 2.3.4 Data analysis

Descriptive statistics were performed to present subject characteristics and distribution of the KAP-HEQ scale scores. To facilitate interpretation, the scale raw scores were all transformed to a scoring range from 0 to 100.

We assessed feasibility by completion rate and completion time (30). We expected a 100% completion rate as the standard. A survey length that can be completed within 10 min is usually considered most feasible for adolescents (22), and within 20 min is feasible for parents. We further assessed the spread of responses across the options to examine any bias to a single option in each item (18). Ordinal-scale items with ≥80% of persons opting for the same response were reviewed for removal.

To establish the internal construct validity of the KAP-HEQ, item-scale correlations of each item and the three KAP scales were computed using the Spearman correlation test. An item should have a significantly positive correlation with its hypothesized scale score (*p*-value < 0.05) to support item convergent validity, and weaker correlations with the other two scale scores to support item discriminant validity. The item-scale correlation was corrected for overlap by excluding the item to be examined from the summation of the scale score.

The reliability of the KAP-HEQ was analyzed in two ways: (i) Internal consistency of the items in each scale was determined using Cronbach’s alpha, with values of ≥0.7 considered the standard for good reliability (31–33); (ii) Test–retest reliability was assessed by intraclass correlation coefficient (ICC), with values < 0.5 indicating poor reliability, 0.5–0.75 indicating moderate reliability, and >0.75 indicating good reliability (34). Paired *t*-tests were performed to assess any significant difference between test and retest scores. As we could not find another generic Chinese KAP instrument, we hypothesized the three KAP scales measure related but distinct constructs and therefore should have higher internal consistency than inter-scale correlations for discriminant validity.

Known-group comparisons were carried out to assess the external criterion validity and sensitivity of the KAP-HEQ in detecting a difference between groups. Four group comparisons were carried out based on (i) FV intake (≥ vs. <4 servings daily), (ii) family role (parents vs. adolescents), (iii) parental education level (≥ vs. <13 years), and (iv) monthly household income (≥ vs. <HKS20,000). The groupings were based on both the theoretical definition and the sample median. A higher KAP Overall or scale score was hypothesized in the former group in each comparison. Two-sample t-tests were used to test the significance of the group differences. Effect size, measured by the mean difference between two groups divided by the standard deviation, was used to assess the sensitivity in detecting a clinically important difference with 0.2–0.49 indicating a small effect, 0.5 to 0.79 a medium effect, and ≥0.8 a large effect (35, 36).

Sensitivity analysis was performed by the target group (adolescents and adult parents) to explore any discrepancies from the results of the combined-group analysis. The same set of psychometric tests including item-scale correlations, internal consistency, test–retest reliability, and known-group comparisons (except family role) was repeated on the two populations. Item-scale correlations and internal consistency were further evaluated by gender of adolescents to explore any potential differences.

The statistical analyses were performed using SPSS version 28.0 (37). All significance tests were two-tailed with statistical significance set at a *p*-value < 0.05.

## 3 Results

Table 1 shows the characteristics of 8 experts and 16 lay persons who evaluated the content validity and 120 persons of the pilot test.

**Table 1:** Characteristics of subjects in content validity evaluation and pilot testing studies.

	Content validity evaluation						Pilot testing			
	Experts (*N* = 8)		Adolescents (*N* = 8)		Parents (*N* = 8)		Adolescents (*N* = 60)		Parents (*N* = 60)	
	n/Mean	%/SD	n/Mean	%/ SD	n/Mean	%/SD	n/Mean	%/SD	n/Mean	%/SD
**Gender**										
Female	4	50.00%	4	50.00%	8	100.00%	33	55.00%	57	95.00%
**Profession**										
Expert in PRO measures	2	25.00%								
Dietitian	1	12.50%								
Primary care doctor	1	12.50%								
Statistician	1	12.50%								
Pediatrician	1	12.50%								
Co-investigator of the qualitative study	2	25.00%								
Age			15.9	±1.9	51.5	±5.8	15.5	±1.9	47.0	±5.3
**Of adolescents**										
12–13			1	12.50%			10	16.70%		
14–15			2	25.00%			22	36.70%		
16–17			4	50.00%			17	28.30%		
18–19			1	12.50%			11	18.30%		
**Marital status of parents**										
Married					6	75.00%			46	76.70%
Single/Divorced/Separated/Widowed					2	25.00%			14	23.30%
**Education level of parents**										
Primary or below					3	37.50%			6	10.00%
Junior Secondary					2	25.00%			24	40.00%
Senior Secondary					0	0.00%			26	43.30%
Tertiary or above					3	37.50%			4	6.70%
**Employment status of parents**										
Unemployed					3	37.50%			30	50.00%
Part-time					1	12.50%			12	20.00%
Full-time					4	50.00%			18	30.00%
**Monthly household income**										
Below $13,500 (Poverty)					3	37.50%			22	36.70%
$13,500–$19,999					2	25.00%			15	25.00%
$20,000–$26,999					1	12.50%			14	23.30%
$27,000 or above (Median or above)					2	25.00%			11	15.00%
**KAP of healthy eating scores**										
Overall							56.6	±7.4	63.1	±9.4
Knowledge							57.7	±15.8	61.4	±14.7
Attitude							53.3	±8.3	59.6	±9.8
Practice							58.8	±11.1	68.3	±12.9

KAP, Knowledge, Attitudes, and Practices; PRO, patient-reported outcome; SD, standard deviation. The KAP scores were transformed to scales with a range from 0 to 100.

### 3.1 Content validity

All expert panelists and lay persons completed the content evaluation of each item of the first draft of the 48-item KAP-HEQ. A summary of the results on content validity is shown in Supplementary Table 1.

#### 3.1.1 Expert panel review

All except five items of KAP-HEQ achieved the standards of CVR (≥0.75) and CVI (≥0.78) on relevance and clarity. Two items had a low CVR of 0.5, one on Knowledge (#10 “Eating vegetables promotes skin health”) and one on Attitudes (#16 “Avoid unhealthy eating habits to maintain health”). Our research team reviewed these two items and decided to retain the Knowledge item, which was evidence-based (38, 39), but to remove the Attitude item since similar content is measured by another item (#17 “Healthy eating habit for a good figure and weight”). Three items (#1, 32, 41) had a CVI of 0.75 on clarity. Our research team reworded these three items as well as a few other items (#4, 13, 15, 17, 20, 22, 28, 31, 38) and the instructions to address the feedback from the panel members. For example, in #38 “Choose food with ‘0% fat/ No added salt/sugar’ or ‘Low in fat/ salt/sugar’ written on the package” was changed to “Choose food with ‘No added’ or ‘Low’ salt/sugar/fat written on the package” to improve the clarity. All panel members agreed that the questionnaire items were comprehensive for the relevant construct, except one member suggested including “bringing lunchbox to school” under Practices. The team considered that the home-prepared lunchbox is covered under #33 “home-prepared meals,” and thus deemed unnecessary to add a separate item for it.

#### 3.1.2 Evaluation by lay persons of the target population

In total, 18 items did not reach the standard of CVR ≥ 0 for importance or CVI ≥ 0.75 for relevance or clarity. Our research team reviewed these items and decided to remove 3 items (#11, 23, and 35) that were not essential, and retain 4 items (#20, 22, 45, and 46) with low CVR but optimal standards of relevance and clarity because the team found them theoretically significant, and reworded 11 items (#5, 6, 7, 9, 19, 21, 27, 28, 29, 30, and 32) to improve clarity. To illustrate, the presentation of the nutrition labels in a Knowledge item (#9) was modified and one response option was removed; two Attitude items #28 and #31 “Perceived ability to eat sufficient fruit/ vegetables” were reworded from negative to positive statements to avoid misunderstanding. For qualitative feedback, one lay person suggested adding a Knowledge item to the daily recommendation of meat intake, but the research team decided this was not appropriate for the purpose of the KAP-HEQ. Another person suggested specifying the size of a bowl of fruit in Attitude item (#28) and commented that it was difficult to recall the frequency of consumption to answer the Practice items. The research team deliberated the comments and decided to make no change to these items since the bowl size is already defined as 300 mL in item #2 and as 2 fist-size in item #37; moreover, recall difficulty is unavoidable in a survey.

Based on the results of the content validity evaluation, 4 items were removed from the KAP-HEQ. The remaining 44 KAP items formed the final draft of questionnaires for the pilot test (see Supplementary Data Sheets 2).

### 3.2 Pilot test on feasibility and psychometrics

#### 3.2.1 Feasibility

In total, 60 parent–adolescent dyads (120 persons) provided consent and completed the final draft of KAP-HEQ online. Each parent and adolescent completed the questionnaire survey independently. All persons completed all items with no missing data; 105 (87.5%) persons completed the survey within 60 min and 15 persons (6 adolescents and 9 parents) took over 1.5 h and up to 6 days to submit the survey results. Excluding these 15 outliers, the valid mean completion time was 9.49 ± 8.71 min (2.95–45.08 min) among the 54 adolescents and 21.32 ± 9.59 min (4.77–58.77 min) among the 51 parents who had to answer 44 additional items on family factors (e.g., parenting style and food parenting practices) and sociodemographics. No single option in the 5-point ordinal scale of the Attitude and Practice items was chosen by ≥80% of persons, implying no systematic bias in these scales.

#### 3.2.2 Construct validity

Table 2 shows the item-scale correlations of each item of KAP-HEQ with each of the three KAP scales using the Spearman correlation test. Most of the items had a significantly positive item-scale correlation (*p* < 0.05) with its hypothesized scale except five items on Knowledge and two on Attitudes (Q1, Q3, Q4, Q8, Q10, Q12b, and Q12j), which achieved the standard on item convergent validity. Over 80% of the items in each KAP scale had a higher correlation with its hypothesized scale than with the other two scales, which achieved the standard on item discriminant validity. Two Knowledge items (Q1 and Q3), three Attitude items (Q12b, Q12j, and Q12l), and three Practice items (Q13a, Q15a, and Q15c) did not meet the standard.

**Table 2:** Item-scale correlations of the KAP scales (*N* = 120).

Item	Scale		
	Knowledge	Attitudes	Practices
**Knowledge**			
Q1	**0.08**	0.09	0.15
Q2	**0.20** ^*^	0.07	0.07
Q3	**−0.05**	0.18	0.27^**^
Q4	**0.18**	0.01	0.10
Q5	**0.34** ^***^	0.16	0.10
Q6	**0.20** ^*^	0.03	0.08
Q7	**0.19** ^*^	0.02	−0.06
Q8	**0.14**	0.08	−0.02
Q9a	**0.23** ^*^	0.13	0.19^*^
Q9b	**0.33** ^***^	0.08	0.11
Q9c	**0.32** ^***^	0.05	0.10
Q9d	0.33***	0.13	0.14
Q9e	0.30**	0.02	0.07
Q10	**0.08**	−0.09	−0.03
**Attitudes**			
Q12a	0.19*	**0.58** ^***^	0.22^*^
Q12b	−0.03	**−0.10**	0.20^*^
Q12c	0.17	**0.47** ^***^	0.12
Q12d	0.30^***^	**0.59** ^***^	0.35^***^
Q12e	0.05	**0.61** ^***^	0.36^***^
Q12f	0.16	**0.29** ^**^	0.23^*^
Q12g	0.13	**0.60** ^***^	0.37^***^
Q12h	0.04	**0.51** ^***^	0.3^***^
Q12i	0.04	**0.53** ^***^	0.36^***^
Q12j	0.16	**0.13**	0.15
Q12k	0.15	**0.60** ^***^	0.46^***^
Q12l	0.16	**0.51** ^***^	0.58^***^
Q12m	0.08	**0.53** ^***^	0.48^***^
Q12n	0.14	**0.58** ^***^	0.17
Q12o	0.11	**0.71** ^***^	0.4^***^
**Practices**			
Q13a	0.18	0.27**	**0.24** ^*^
Q13b	0.03	0.26**	**0.45** ^***^
Q13c	0.03	0.42***	**0.60** ^***^
Q13d	0.18^*^	0.37^***^	**0.62** ^***^
Q13e	0.07	0.17	**0.38** ^***^
Q14a	0.08	0.3^**^	**0.42** ^***^
Q14b	0.13	0.29^**^	**0.36** ^***^
Q14c	0.23*	0.26^**^	**0.47** ^***^
Q14d	0.14	0.35^***^	**0.58** ^***^
Q15a	0.16	0.35^***^	**0.28** ^**^
Q15b	0.18	0.30^**^	**0.41** ^***^
Q15c	0.13	0.33^***^	**0.32** ^***^
Q15d	0.18*	0.32^***^	**0.57** ^***^
Q15e	0.07	0.27^**^	**0.39** ^***^
Q15f	0.06	0.23^*^	**0.3** ^***^

All coefficients in bold were corrected for overlap by excluding the item score from the scale score calculation.

^*^p-value < 0.05; ^**^p-value < 0.01; ^***^p-value < 0.001 by Spearman correlation test.

#### 3.2.3 Internal consistency and test–retest reliability

Table 3 shows the internal consistency and inter-scale correlations of the KAP scales. The Overall, Attitude, and Practice scales had a good internal consistency with Cronbach’s alpha above 0.7. The Knowledge scale had a modest internal consistency (α = 0.54). The alpha coefficients of each KAP scale did not have much variation if any of the items were deleted. All the scales had a higher internal consistency than the inter-scale correlations, indicating each scale measured a distinct construct.

**Table 3:** Internal consistency and inter-scale correlations of KAP scales (*N* = 120).

Scale	Cronbach’s alpha	Inter-scale correlation	
		Attitudes	Practices
Overall	0.87		
Knowledge	0.54	0.23^*^	0.21^*^
Attitudes	0.84		0.55^***^
Practices	0.81		

^*^p-value < 0.05; ^***^p-value < 0.001 by the Spearman correlation test.

Both the Overall and individual scale scores had good test–retest reliability with ICC above 0.75 except for the Knowledge scale (ICC = 0.58; Table 4). No significant difference was found in the mean KAP scores using the paired t-test with small effect sizes (d < |0.2|) between the test and retest.

**Table 4:** Test–retest reliability of the KAP scales (*N* = 60).

Scale	ICC	Mean (T0)	Mean (T1)	P-value of paired t-test	Cohen’s d effect size
Overall	0.86	62.71	63.64	0.33	−0.13
Knowledge	0.58	63.02	65.21	0.33	−0.13
Attitudes	0.84	60.50	61.25	0.59	−0.07
Practices	0.84	64.61	64.47	0.91	0.01

ICC, intraclass correlation coefficient; T0, first test; T1, second test in 2 weeks.

#### 3.2.4 Known-group comparisons

The mean scores of KAP were compared by known groups classified by FV intake, family role, parental education level, and household income (Table 5). The differences found in all the group comparisons followed the hypothesized trend except the Attitude score by household income (mean difference = −1.60, *p* = 0.56). Subjects who consumed at least 4 servings of FV daily (compared to <4 servings of FV) had higher scores in all KAP scales (mean difference = 6.47–10.94, *p* < 0.05). Parents had better Overall, Attitude, and Practice scores (mean difference = 7.26–9.50, *p* < 0.001) than adolescents. Dyads with parental education of 13 years or above had higher Overall, Knowledge, and Attitude scores (mean difference = 4.14–5.73, *p* < 0.05), whereas those with a monthly household income of HK$20,000 or above had better Knowledge scores (mean difference = 9.00, *p* < 0.01).

**Table 5:** Known-group comparison of the KAP scales (*N* = 120).

	Mean difference	*P*-value	Cohen’s effect size
	**FV intake**		
	**[≥4 servings (*n* = 36) vs. <4 servings daily (*n* = 84)]**		
Overall^a^	8.66^***^	<0.001	0.93
Knowledge	6.47^*^	0.03	0.43
Attitudes	8.58^**^	0.00	0.66
Practices^a^	10.94^***^	<0.001	0.90
	**Family role**		
	**[Parents (*n* = 60) vs. Adolescents (*n* = 60)]**		
Overall	7.26^***^	<0.001	0.77
Knowledge	3.65	0.19	0.24
Attitudes	8.64^***^	<0.001	0.67
Practices	9.50^***^	<0.001	0.79
	**Parental education level**		
	**[≥13 years (*n* = 60) vs. <13 years (*n* = 60)]**		
Overall	4.14^*^	0.02	0.42
Knowledge	5.73^*^	0.04	0.38
Attitudes	4.97^*^	0.04	0.37
Practices	1.72	0.47	0.13
	**Monthly household income**		
	**[≥$20,000 (*n* = 46) vs. <$20,000 (*n* = 74)]**		
Overall	3.38	0.07	0.34
Knowledge	9.00^**^	0.00	0.61
Attitudes	−1.60	0.56	−0.12
Practices	2.73	0.26	0.21

^a^Corrected for overlap by the exclusion of the item score of FV intake from the scale score calculation. ^*^p-value < 0.05; ^**^p-value < 0.01; ^***^p-value < 0.001 by the Spearman correlation test.

Significant differences in each KAP score were found in at least two group comparisons with small to large effect sizes, supporting sensitivity. The Overall and Attitude scores were sensitive in differentiating subjects by FV intake (d = 0.93 and 0.66), family role (d = 0.77 and 0.67), and parental education level (d = 0.42 and 0.37). The Knowledge score was sensitive in detecting a difference between groups by FV intake, parental education level, and household income (d = 0.43, 0.38, and 0.61, respectively). The Practice score was sensitive in detecting a difference between groups by FV intake (d = 0.90) and family role (d = 0.79).

#### 3.2.5 Sensitivity analysis by target group

The results of the sensitivity analysis by adolescents and parents and by gender of adolescents are presented in Supplementary Tables 2–8. Most items of the KAP-HEQ achieved the standard of item convergent validity and item discriminant validity in adolescents (65.9% and 86.4%) and in parents (77.3% and 70.5%). The Overall, Attitude, and Practice scales had a good internal consistency (α > 0.7), and the Knowledge scale had a modest internal consistency (α = 0.56 and 0.52) in both groups. Both the Overall and individual scale scores had good test–retest reliability (ICC > 0.75) in both groups except for the Knowledge and Practice scales in adolescents (ICC = 0.43 and 0.60). Differences in the group comparisons followed the hypothesized trend in parents but not by parental education or household income in adolescents. Differences in some KAP scale scores by FV intake were found in both groups (mean difference = 4.39–10.96, *p* < 0.05), whereas differences by parental education and household income were only found in parents (mean difference = 7.06–14.2, *p* < 0.05). The Overall and Practice scores were sensitive in detecting a difference between adolescents by FV intake (d = 0.56 and 0.83), whereas the Overall and all scale scores were sensitive in differentiating parents by FV intake (d = 0.60–0.99), parental education (d = 0.57–0.81), and/or household income (d = 0.68–1.09), with medium to large effect sizes.

Female and male adolescents had similar KAP scale scores except higher Practice scores in female adolescents (61.46 vs. 55.43, *p* < 0.05). More KAP-HEQ items achieved the item convergent and discriminant validity standards in male adolescents (61.4% and 84.1%) than in female (40.9% and 77.3%) adolescents. The results on the internal consistency of the scales were similar between genders (α > 0.7 for the Overall, Attitude, and Practice scales, α = 0.45 in female adolescents, and α = 0.67 in male adolescents for the Knowledge scale).

## 4 Discussion

The new KAP-HEQ tested in our study is the first generic measure of KAP of healthy eating applicable to both adults and adolescents in Chinese culture. The content validity of the final 44-item measure was supported by evaluations by both experts and lay persons. It was feasible and acceptable for online self-completion by adolescents and their adult parents within a short completion time of 10–15 min. The final KAP-HEQ demonstrated good construct validity and acceptable reliability, overall and at the scale level.

Many items of the KAP-HEQ are specific to the local Chinese culture and guided by the results of our earlier qualitative study (25, 26). For instance, the items on the definition of one serving of FV (item Q2) and the examples of vegetables (item Q8) were included to address these knowledge gaps found in the qualitative study. We also included items on the misconceptions of low susceptibility to health consequences at a young age (item Q12f) and health maintenance solely by exercise (item Q12j), which were found in the dyad interviews, especially among the adolescents.

We observed a relatively low rating on importance among the lay persons where 16 KAP items were rated essential by less than half of the raters. They might perceive the knowledge of healthy snacks and eating-out options (#5–7) as unimportant because they rarely consume these food items; some might not regard meeting the recommended servings of FV, salt, and sugar as necessary, and hence rated low importance on self-efficacy in following these eating habits (#28–32). The results could reflect the limitations in the knowledge and scope of healthy eating among the general population (25, 40, 41).

The data from 120 individuals from 60 dyads in the pilot study were pooled for analysis of construct validity, reliability, and sensitivity, as the KAP-HEQ is intended to be generic, i.e., applicable to all age groups. The KAP scales had satisfactory construct validity in terms of convergent and discriminant validity—almost 80% of items had scaling success, i.e., a significantly positive correlation with the hypothesized scale that was higher than or similar to those with other scales (difference > −0.05). Among the 9 items that did not have scaling success, 5 were Knowledge items that represented the gaps identified in our previous qualitative study, which are dietary recommendations on FV (Q1), salt (Q3), and sugar intake (Q4), examples of vegetables (Q8), and interpretation of nutrition labels (Q10). Items Q1 and Q3 had a higher correlation with Practice than Knowledge scale score, possibly because respondents who knew the recommended intake of FV and salt tended to eat more healthily. Two Attitude items were intended to assess common misconceptions: one about the inferior taste of healthy food (Q12b) and the other about achieving health maintenance solely through exercise (Q12j). Hence, these items were often negatively/wrongly answered by the respondents. Having taste preference toward healthy food (Q12b) or against food with high fat, sugar, or salt (Q12l) favored healthier practices, which could explain why these items correlated more with the Practice than the Attitude scale score. The Practice item of using nutrition claims on the package in food decision (Q15a) might be more common among those with positive attitudes toward healthy eating, explaining its higher correlation with Attitude than Practice scale score. Taking the evaluation results of content validity into account, we think these items should be retained in the proposed scales, but further studies on larger samples should be carried out to determine the validity of items Q3 and Q12b that had negative correlations with their respective scales.

The reliability results of this KAP-HEQ were comparable to those reported for existing dietary KAP measures in the literature. Our instrument showed good reliability in general (Cronbach’s α = 0.81–0.88, ICC = 0.84–0.86) although the Knowledge scale had a relatively low internal and test–retest reliability (Cronbach’s α = 0.54, ICC = 0.58). The reliability of dietary KAP questionnaires reported in the literature varied widely with Cronbach’s α ranging from 0.39 to 0.89 and ICC ranging from 0.07 to 0.96 (10, 13, 21). A low Cronbach’s α may be partly related to a small number of items and partly caused by the heterogeneity of the items in the scale (32). The latter could explain the relatively low internal reliability found in the Knowledge scale since the items measure different scopes (e.g., daily recommendation, effect on health) and depths of food knowledge (e.g., different food categories of FV, sugar, and salt).

The results of known-group comparisons further supported the construct validity of the KAP-HEQ, Overall, and by the individual KAP scales. The KAP-HEQ also showed sensitivity in differentiating groups by FV intake, family role, parental education, and household income, with small to large effect sizes, suggesting its potential application in differentiating people between high and low levels of KAP of healthy eating.

The sensitivity analysis performed by the target group showed some variations between adolescents and adult parents, but most of the evaluation standards were achieved. Some suboptimal outcomes included fewer items with scaling success in both groups (65.9 and 70.5% vs. 79.5%), lower test–retest reliability on Knowledge and Practice scales in adolescents than in parents (ICC = 0.43 and 0.60 vs. 0.78 and 0.91) and no score differences observed in group comparisons by parental education and household income in adolescents. It should be noted that the sample size was 60 persons (and 30 persons for test–retest reliability) in the subgroup analysis, which reduced the power of the findings (42). On the other hand, the dietary knowledge, attitudes, and practices in adolescents are fast-changing during the transition from children to adults, which could interfere with the test–retest evaluation results. The influence of social modeling of healthy eating from family, friends, school, and media on adolescents (43) may explain the weak association among parental education, household income, and KAP scale scores in the group comparisons. In general, the results by target group were comparable to the combined-group findings, supporting the appropriateness of analyzing the combined data from both adolescents and adult parents in this pilot test.

The psychometric tests among female and male adolescents showed similar results. There was a lower scaling success rate in female adolescents (40.9% vs. 61.4%). In particular, the scaling success of the items on perceived susceptibility to chronic disease (Q12f) and health maintenance solely by exercise (Q12j) was found in male adolescents only. This could be related to the younger ages of female adolescents whose cognition and behaviors are less developed than the older male adolescents. Female adolescents might also be more receptive to external influence in the social and physical environments (44), which interferes with their internalization of KAP of healthy eating. We must point out that the subgroup sample sizes were small (33 female adolescents and 27 male adolescents), and the results should be interpreted with caution.

### 4.1 Implications of findings

With our pilot test data supporting the validity, reliability, and sensitivity of the new KAP-HEQ for Chinese populations, we can apply it to assess the level of KAP among adolescents and adults to identify gaps for intervention. It may also be used to monitor changes over time or after interventions by repeated measurement since it is acceptable with a short completion time and has good test–retest reliability. Further study is required to evaluate its psychometrics properties among male adults and confirm its responsiveness.

### 4.2 Strengths and limitations

This study had several strengths. First, we included both experts and lay parents and adolescents to evaluate the content validity in the development stage to ensure that the KAP-HEQ is not only theoretically sound but also relevant to the target population. Second, the inclusion of community-dwelling parents and adolescents supports the KAP-HEQ to be a generic measure applicable to both adolescents and adults with different health statuses. Third, there are only 44 items in this KAP-HEQ, which is much shorter than the existing KAP instruments with increased acceptance among the respondents. Fourth, the questionnaires can be administered on a digital platform providing an efficient way to collect data without location barriers. A digital platform also reduces human error in data entry, which saves a significant amount of time from the researchers’ perspective.

Some limitations were also identified. The sample size of 120 persons was relatively small, and the sensitivity analysis by target group should be further assessed with at least 100 persons from each group (42). The subjects were mostly recruited from the cohort families of an existing health empowerment study whose results might not be representative of all Chinese people. The adult parents were dominated by female and further exploration is warranted to support its application on male adults. We evaluated the content validity through a quantitative survey but did not carry out qualitative cognitive debriefing (16) to minimize respondent burden, which limited the evaluation of how respondents interpreted the items.

## 5 Conclusion

The KAP-HEQ has shown good validity, reliability, and sensitivity in assessing the knowledge, attitudes, and practices of healthy eating among Chinese adolescents and adult parents in this pilot study. Further studies with the inclusion of Chinese adults and adolescents of both genders from different socioeconomic backgrounds and in other parts of the world are required to confirm the validity and psychometric properties of this KAP-HEQ across different Chinese populations. Longitudinal studies are also required to establish its responsiveness in detecting changes after interventions. With additional studies supporting our findings, the KAP-HEQ can be applied to identify the KAP status and gaps in order to inform the design of healthy eating promotion interventions. It may also be used to evaluate the effectiveness of healthy eating interventions.

## Data availability statement

The raw data supporting the conclusions of this article will be made available by the authors, without undue reservation.

## Ethics statement

The studies involving humans were approved by Institutional Review Board of the University of Hong Kong/Hospital Authority Hong Kong West Cluster. The studies were conducted in accordance with the local legislation and institutional requirements. Written informed consent for participation in this study was provided by the participants’ legal guardians/next of kin.

## Author contributions

KL: Conceptualization, Data curation, Formal analysis, Funding acquisition, Investigation, Methodology, Project administration, Writing – original draft, Writing – review & editing. JC: Funding acquisition, Writing – review & editing. K-SS: Funding acquisition, Writing – review & editing. JT: Data curation, Funding acquisition, Supervision, Writing – review & editing. PI: Funding acquisition, Writing – review & editing. CW: Funding acquisition, Writing – review & editing. CL: Conceptualization, Data curation, Funding acquisition, Investigation, Methodology, Supervision, Writing – review & editing.

## References

[1] Knuppel A Papier K Key TJ Travis RC. EAT-lancet score and major health outcomes: the EPIC-Oxford study. Lancet. (2019) 394:213–4. doi: 10.1016/S0140-6736(19)31236-X, PMID: 31235280

[2] Wang DD Li Y Bhupathiraju SN Rosner BA Sun Q Giovannucci EL. Fruit and vegetable intake and mortality: results from 2 prospective cohort studies of US men and women and a Meta-analysis of 26 cohort studies. Circulation. (2021) 143:1642–54. doi: 10.1161/CIRCULATIONAHA.120.048996 33641343 PMC8084888

[3] GBD 2017 Diet Collaborators. Health effects of dietary risks in 195 countries, 1990-2017: a systematic analysis for the global burden of disease study 2017. Lancet. (2019) 393:1958–72. doi: 10.1016/S0140-6736(19)30041-8 30954305 PMC6899507

[4] Centre for Health Protection. Number of Deaths by Leading Causes of Death, 2001-2019 Hong Kong: Department of Health, HKSAR; (2022). Available at: https://www.chp.gov.hk/en/statistics/data/10/27/380.html.

[5] Centre for Health Protection. Report of population health survey 2020–22 (part I). Hong Kong: Department of Health, HKSAR (2023).

[6] World Health Organization. Advocacy, communication and social mobilization for TB control: A guide to developing knowledge, attitude and practice surveys. Geneva: World Health Organization (2008).

[7] Marías Y Glasauer P. Guidelines for assessing nutrition-related knowledge, attitudes and practices Food and agriculture Organization of the United Nations (Rome: FAO) (2014).

[8] Beech BM Rice R Myers L Johnson C Nicklas TA. Knowledge, attitudes, and practices related to fruit and vegetable consumption of high school students. J Adolesc Health. (1999) 24:244–50. doi: 10.1016/S1054-139X(98)00108-6 10227343

[9] Liu H He S Long H Cao X Zhang Z Zhang Z. Knowledge, attitudes and practices related to WG consumption among college students: a cross-sectional study in Chongqing, China. Public Health. (2021) 190:37–41. doi: 10.1016/j.puhe.2020.10.022, PMID: 33338901

[10] Chau PH Leung AY Li HL Sea M Chan R Woo J. Development and validation of Chinese health literacy scale for low salt consumption-Hong Kong population (CHLSalt-HK). PLoS One. (2015) 10:e0132303. doi: 10.1371/journal.pone.0132303, PMID: 26148008 PMC4492982

[11] Zeng D Fang ZL Qin L Yu AQ Ren YB Xue BY. Evaluation for the effects of nutritional education on Chinese elite male young soccer players: the application of adjusted dietary balance index (DBI). J Exerc Sci Fit. (2020) 18:1–6. doi: 10.1016/j.jesf.2019.08.004, PMID: 31641361 PMC6796798

[12] Anand T Ingle GK Meena GS Kishore J Yadav S. Effect of life skills training on dietary behavior of school adolescents in Delhi: a nonrandomized interventional study. Asia Pac J Public Health. (2015) 27:Np1616. doi: 10.1177/1010539513486922 23666837

[13] CC H YS C YM C MT MN. Development and validation of knowledge, attitude and practice on healthy lifestyle questionnaire (KAP-HLQ) for Malaysian adolescents. J Nutr Health Sci. (2015) 2:407. doi: 10.15744/2393-9060.2.407

[14] Ul Haq I Mariyam Z Li M Huang X Jiang P Zeb F. A comparative study of nutritional status, knowledge attitude and practices (KAP) and dietary intake between international and Chinese students in Nanjing, China. Int J Environ Res Public Health. (2018) 15:910. doi: 10.3390/ijerph15091910, PMID: 30177588 PMC6165259

[15] Heale R Twycross A. Validity and reliability in quantitative studies. Evid Based Nurs. (2015) 18:66–7. doi: 10.1136/eb-2015-102129 25979629

[16] Zamanzadeh V Ghahramanian A Rassouli M Abbaszadeh A Alavi-Majd H Nikanfar AR. Design and implementation content validity study: development of an instrument for measuring patient-centered communication. J Caring Sci. (2015) 4:165–78. doi: 10.15171/jcs.2015.017, PMID: 26161370 PMC4484991

[17] Lawshe CH. A quantitative approach to content validity. Pers Psychol. (1975) 28:563–75. doi: 10.1111/j.1744-6570.1975.tb01393.x

[18] Rattray J Jones MC. Essential elements of questionnaire design and development. J Clin Nurs. (2007) 16:234–43. doi: 10.1111/j.1365-2702.2006.01573.x 17239058

[19] DeVon HA Block ME Moyle-Wright P Ernst DM Hayden SJ Lazzara DJ. A psychometric toolbox for testing validity and reliability. J Nurs Scholarsh. (2007) 39:155–64. doi: 10.1111/j.1547-5069.2007.00161.x 17535316

[20] Rönkkö M Cho E. An updated guideline for assessing discriminant validity. Organ Res Methods. (2022) 25:6–14. doi: 10.1177/1094428120968614

[21] Moitra P Verma P Madan J. Development and validation of a questionnaire measuring knowledge, attitudes, and practices (KAP) to healthy eating and activity patterns in school children (HEAPS). Nutr Health. (2021) 27:199–209. doi: 10.1177/0260106020982356, PMID: 33522877

[22] Omrani A Wakefield-Scurr J Smith J Brown N. Survey development for adolescents aged 11–16 years: a developmental science based guide. Adolesc Res Rev. (2019) 4:329–40. doi: 10.1007/s40894-018-0089-0

[23] Wang D Shi Y Chang C Stewart D Ji Y Wang Y. Knowledge, attitudes and behaviour regarding nutrition and dietary intake of seventh-grade students in rural areas of mi Yun County, Beijing, China. Environ Health Prev Med. (2014) 19:179–86. doi: 10.1007/s12199-013-0372-4, PMID: 24347467 PMC4019754

[24] Liu T Su X Li N Sun J Ma G Zhu W. Development and validation of a food and nutrition literacy questionnaire for Chinese school-age children. PLoS One. (2021) 16:e0244197. doi: 10.1371/journal.pone.0244197, PMID: 33406105 PMC7787443

[25] Liu KSN Chen JY Sun KS Tsang JPY Ip P Lam CLK. Adolescent knowledge, attitudes and practices of healthy eating: findings of qualitative interviews among Hong Kong families. Nutrients. (2022) 14:857. doi: 10.3390/nu14142857 35889813 PMC9316895

[26] Liu KSN Chen JY Sun KS Tsang JPY Ip P Lam CLK. Family facilitators of, barriers to and strategies for healthy eating among Chinese adolescents: qualitative interviews with parent-adolescent dyads. Nutrients. (2023) 15:651. doi: 10.3390/nu15030651 36771358 PMC9920773

[27] Polit DF Beck CT Owen SV. Is the CVI an acceptable indicator of content validity? Appraisal and recommendations. Res Nurs Health. (2007) 30:459–67. doi: 10.1002/nur.20199, PMID: 17654487

[28] Mokkink LB Prinsen C Patrick DL Alonso J Bouter LM De Vet H. COSMIN Study Design checklist for Patient-reported outcome measurement instruments (2019); 1-32. Available at: https://www.cosmin.nl/wp-content/uploads/COSMIN-study-designing-checklist_final.pdf#.

[29] Fung CS Yu EY Guo VY Wong CK Kung K Ho SY. Development of a health empowerment Programme to improve the health of working poor families: protocol for a prospective cohort study in Hong Kong. BMJ Open. (2016) 6:e010015. doi: 10.1136/bmjopen-2015-010015, PMID: 26842271 PMC4746471

[30] Tafforeau J Cobo M Tolonen H Scheidt-Nave C Tinto A. Guidelines for the development and criteria for the adoption of health survey instruments (2005). Available at: https://ec.europa.eu/health/ph_information/dissemination/reporting/healthsurveys_en.pdf.

[31] Nunnally JC. Psychometric theory. 2nd ed. United States: McGraw-Hill (1978).

[32] Tavakol M Dennick R. Making sense of Cronbach’s alpha. Int J Med Educ. (2011) 2:53–5. doi: 10.5116/ijme.4dfb.8dfd, PMID: 28029643 PMC4205511

[33] Taber KS. The use of Cronbach’s alpha when developing and reporting research instruments in science education. Res Sci Educ. (2018) 48:1273–96. doi: 10.1007/s11165-016-9602-2

[34] Koo TK Li MY. A guideline of selecting and reporting Intraclass correlation coefficients for reliability research. J Chiropr Med. (2016) 15:155–63. doi: 10.1016/j.jcm.2016.02.012, PMID: 27330520 PMC4913118

[35] Cohen J. Statistical power analysis for the behavioral sciences. New York: Routledge (2013).

[36] Cohen J. A power primer. Psychol Bull. (1992) 112:155–9. doi: 10.1037/0033-2909.112.1.155 19565683

[37] Corp IBM. IBM SPSS statistics for windows, version 28.0. Armonk, NY: IBM Corp (2021).

[38] Michalak M Pierzak M Kręcisz B Suliga E. Bioactive compounds for skin health: a review. Nutrients. (2021) 13:203. doi: 10.3390/nu13010203, PMID: 33445474 PMC7827176

[39] Evans JA Johnson EJ. The role of phytonutrients in skin health. Nutrients. (2010) 2:903–28. doi: 10.3390/nu2080903, PMID: 22254062 PMC3257702

[40] Han B Li C Zhou Y Zhang M Zhao Y Zhao T. Association of Salt-Reduction Knowledge and Behaviors and salt intake in Chinese population. Front Public Health. (2022) 10:872299. doi: 10.3389/fpubh.2022.872299, PMID: 35509508 PMC9058069

[41] Rooney C McKinley MC Appleton KM Young IS McGrath AJ Draffin CR. How much is ‘5-a-day’? A qualitative investigation into consumer understanding of fruit and vegetable intake guidelines. J Hum Nutr Diet. (2017) 30:105–13. doi: 10.1111/jhn.12393, PMID: 27334026

[42] Anthoine E Moret L Regnault A Sébille V Hardouin JB. Sample size used to validate a scale: a review of publications on newly-developed patient reported outcomes measures. Health Qual Life Outcomes. (2014) 12:176. doi: 10.1186/s12955-014-0176-2 25492701 PMC4275948

[43] Cruwys T Bevelander KE Hermans RC. Social modeling of eating: a review of when and why social influence affects food intake and choice. Appetite. (2015) 86:3–18. doi: 10.1016/j.appet.2014.08.035 25174571

[44] Spencer RA Rehman L Kirk SF. Understanding gender norms, nutrition, and physical activity in adolescent girls: a scoping review. Int J Behav Nutr Phys Act. (2015) 12:6. doi: 10.1186/s12966-015-0166-8 25616739 PMC4310022

