# Supplementary figures and images for: The Knowledge, Attitudes, and Practices of Healthy Eating Questionnaire: a pilot validation study in Chinese families
Source: Front Public Health. 2024 Jul 17;12:1355638. doi: 10.3389/fpubh.2024.1355638 (PMC11288980; doi:10.3389/fpubh.2024.1355638)

**Supplementary Data Sheet 1.** Evaluation form for expert review on the first draft questionnaire


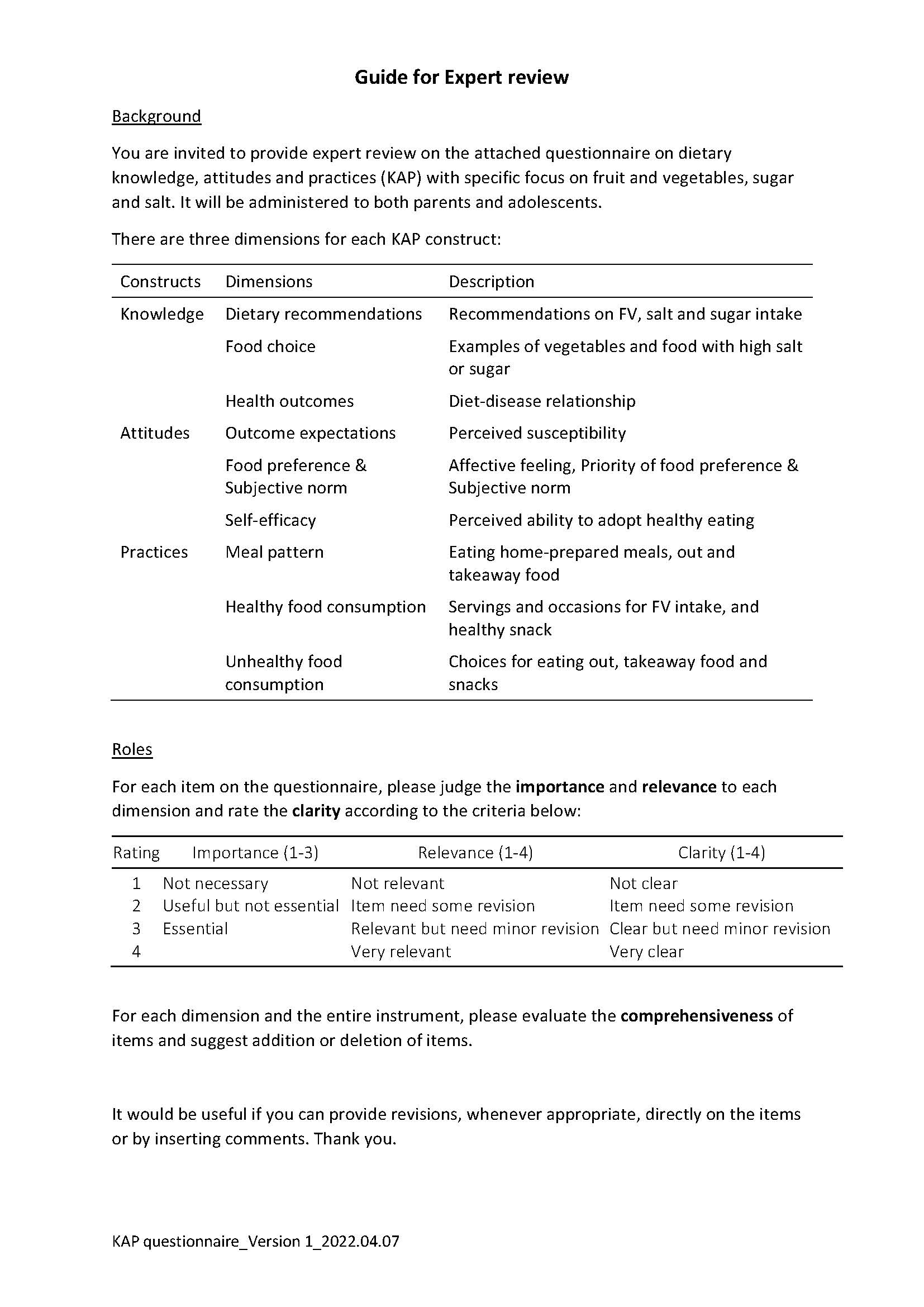


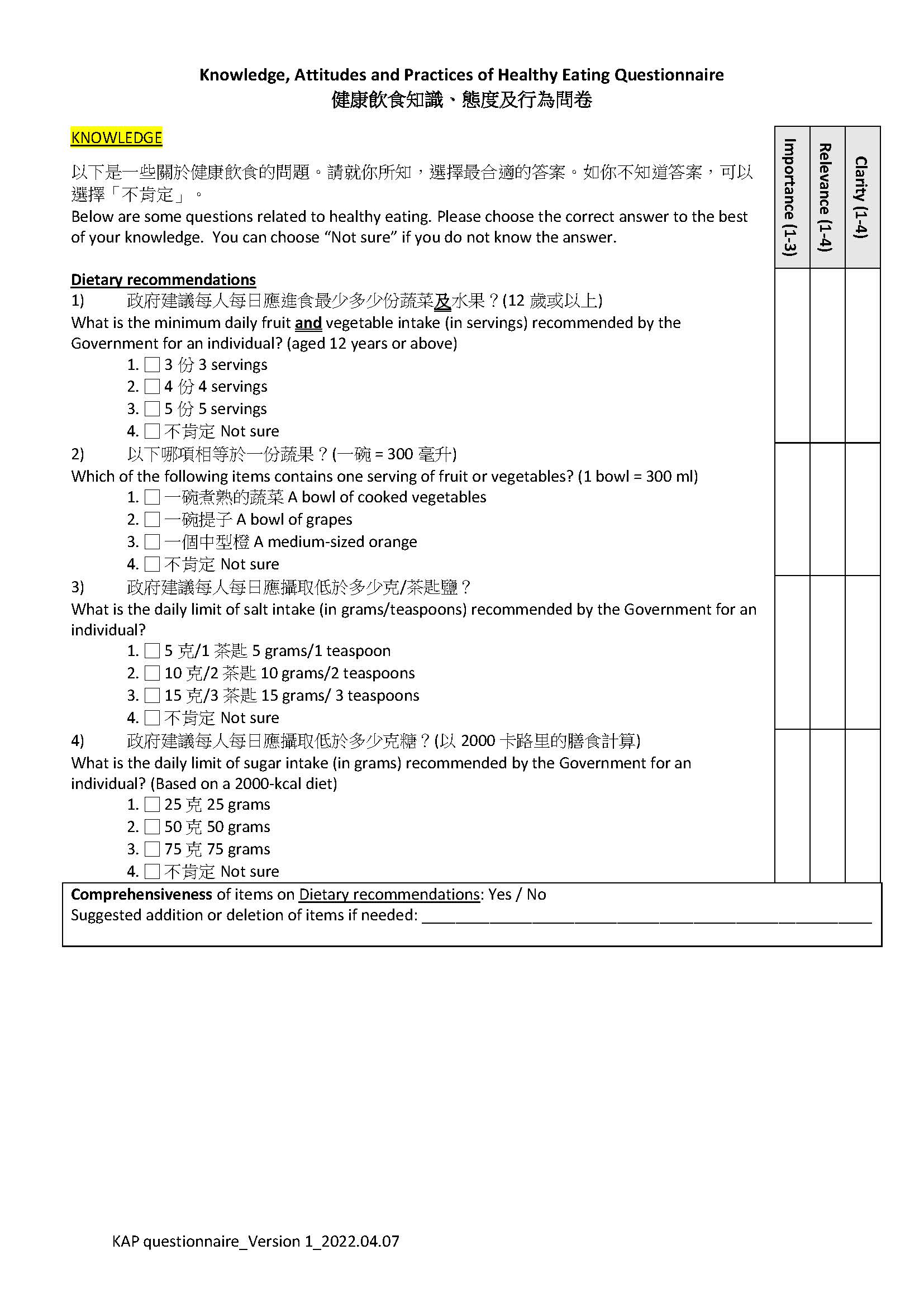

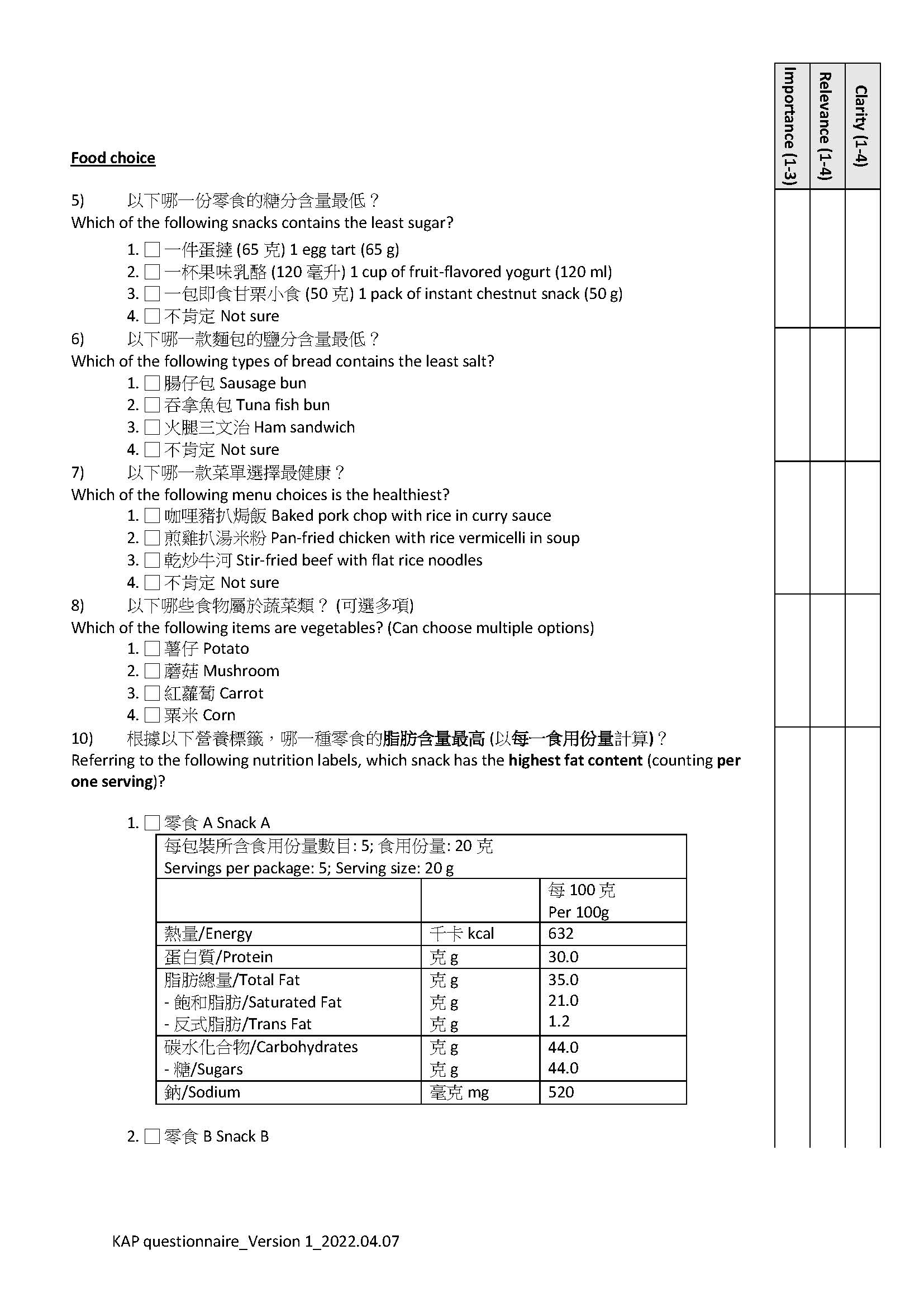

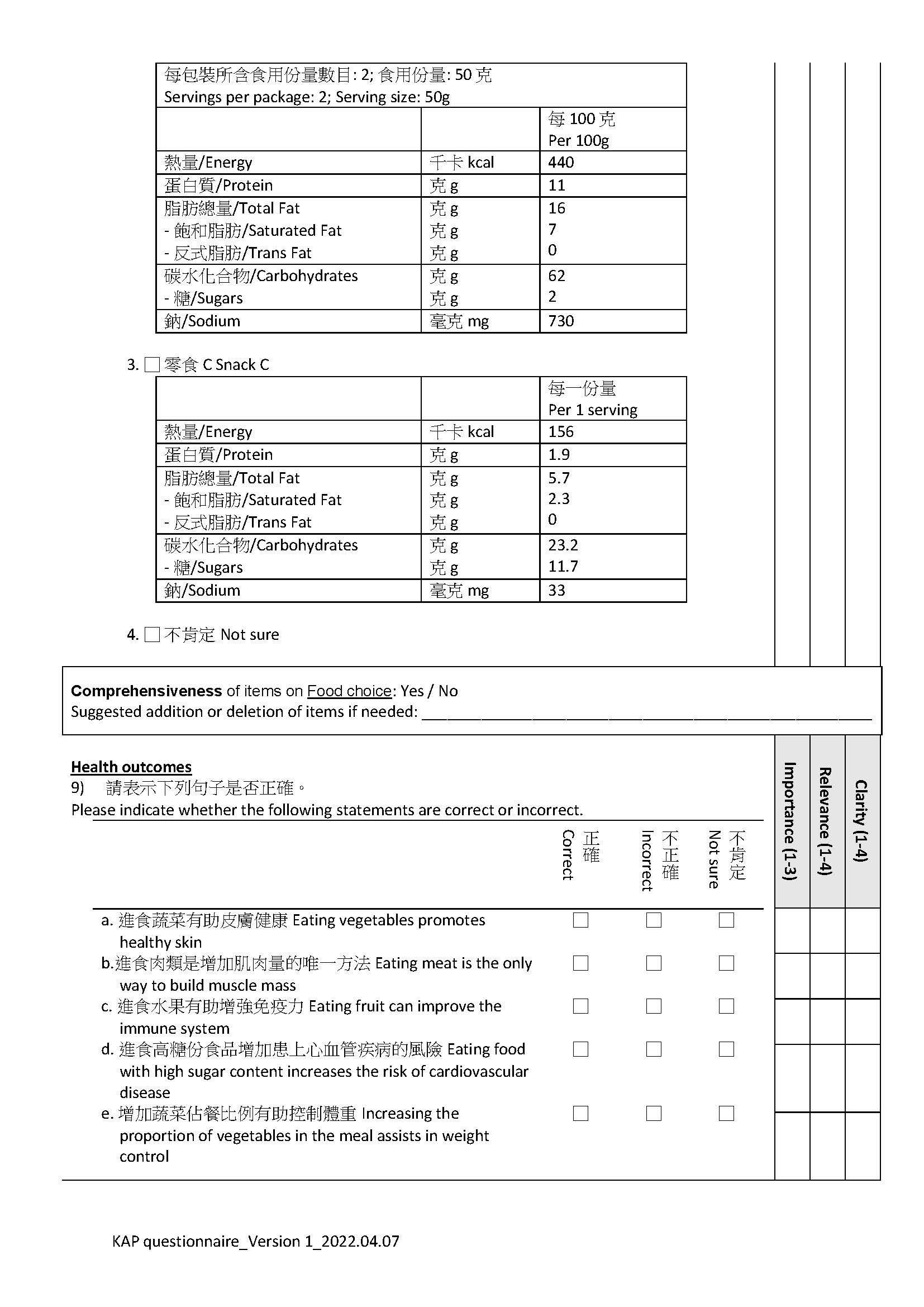

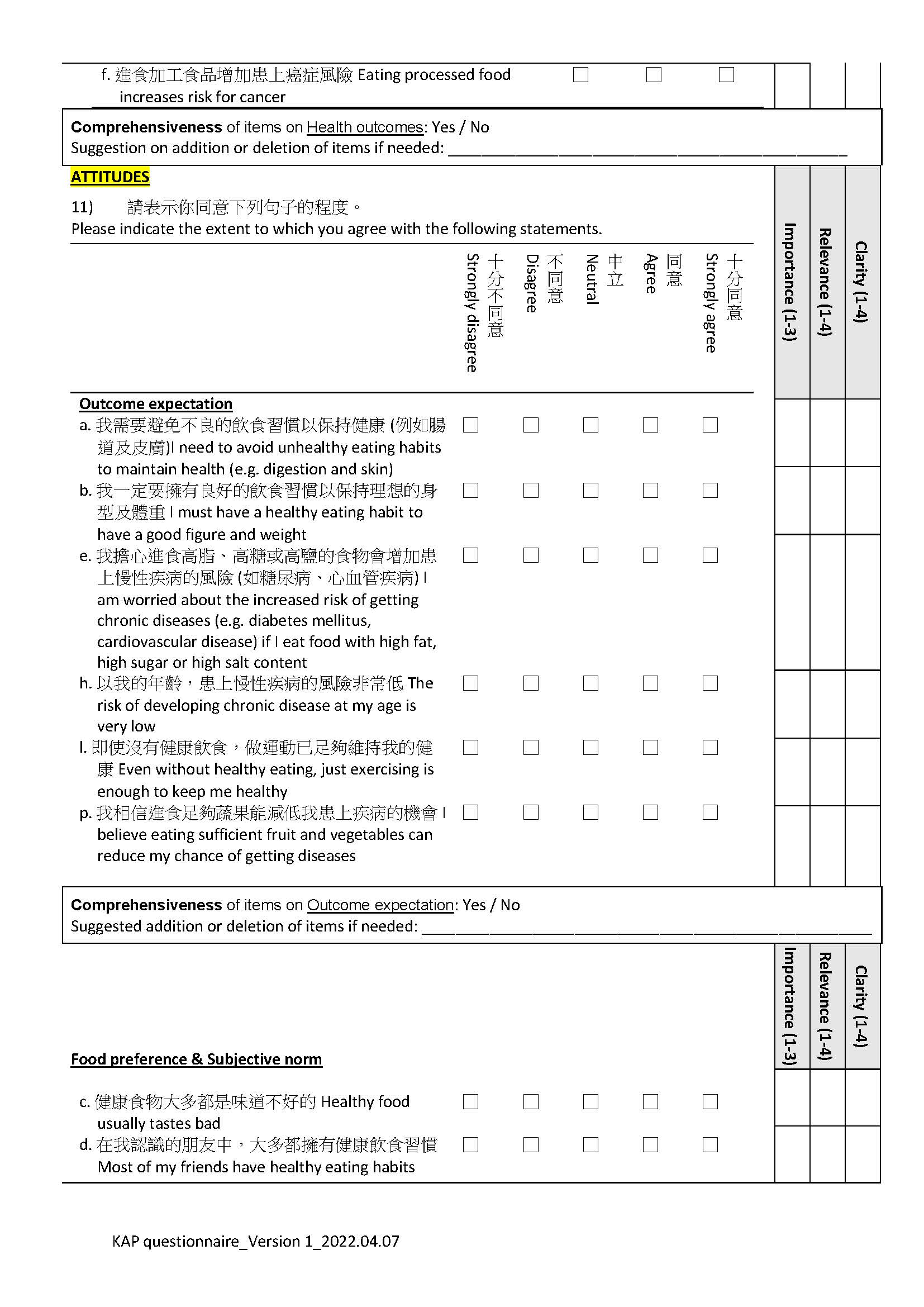

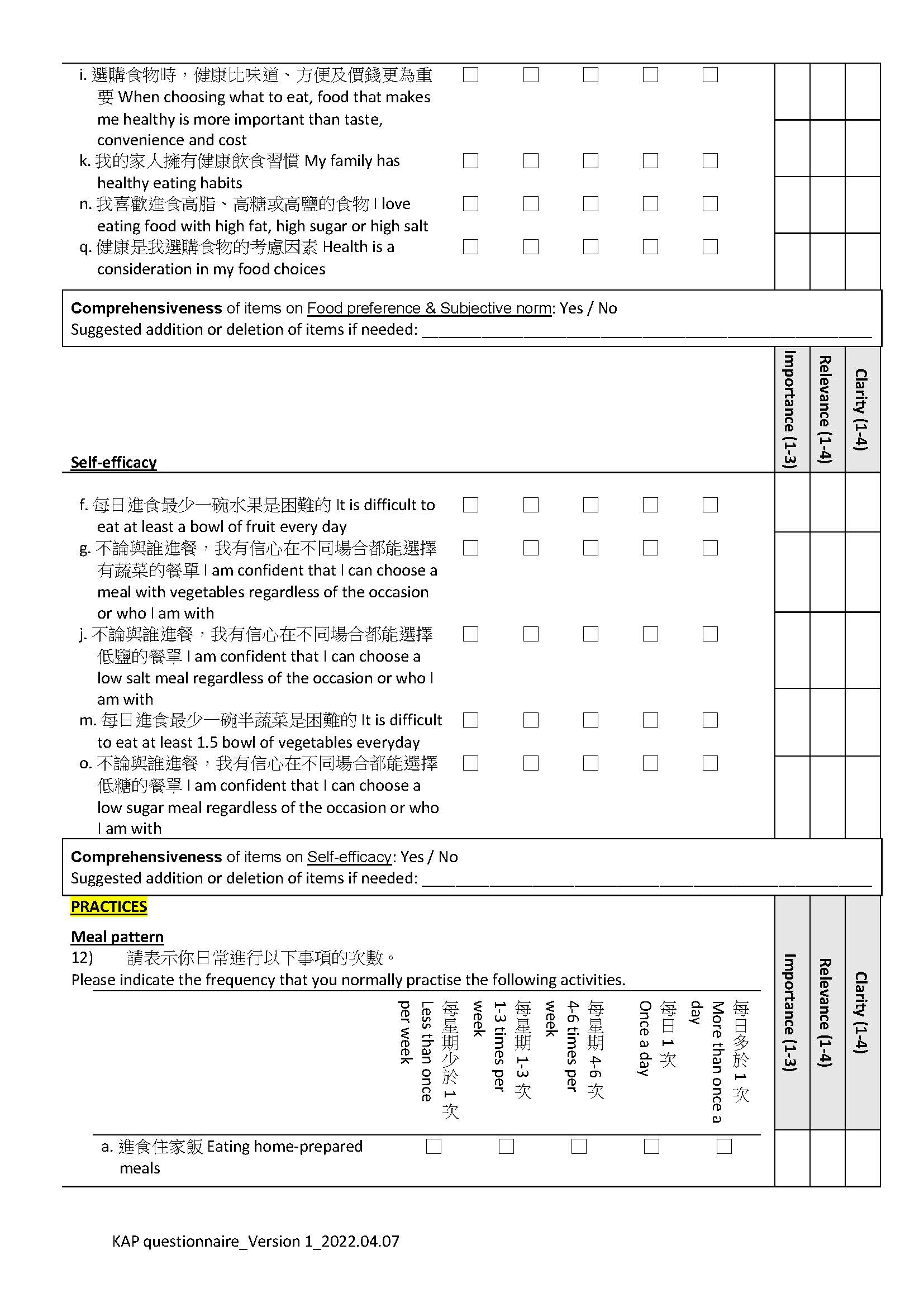

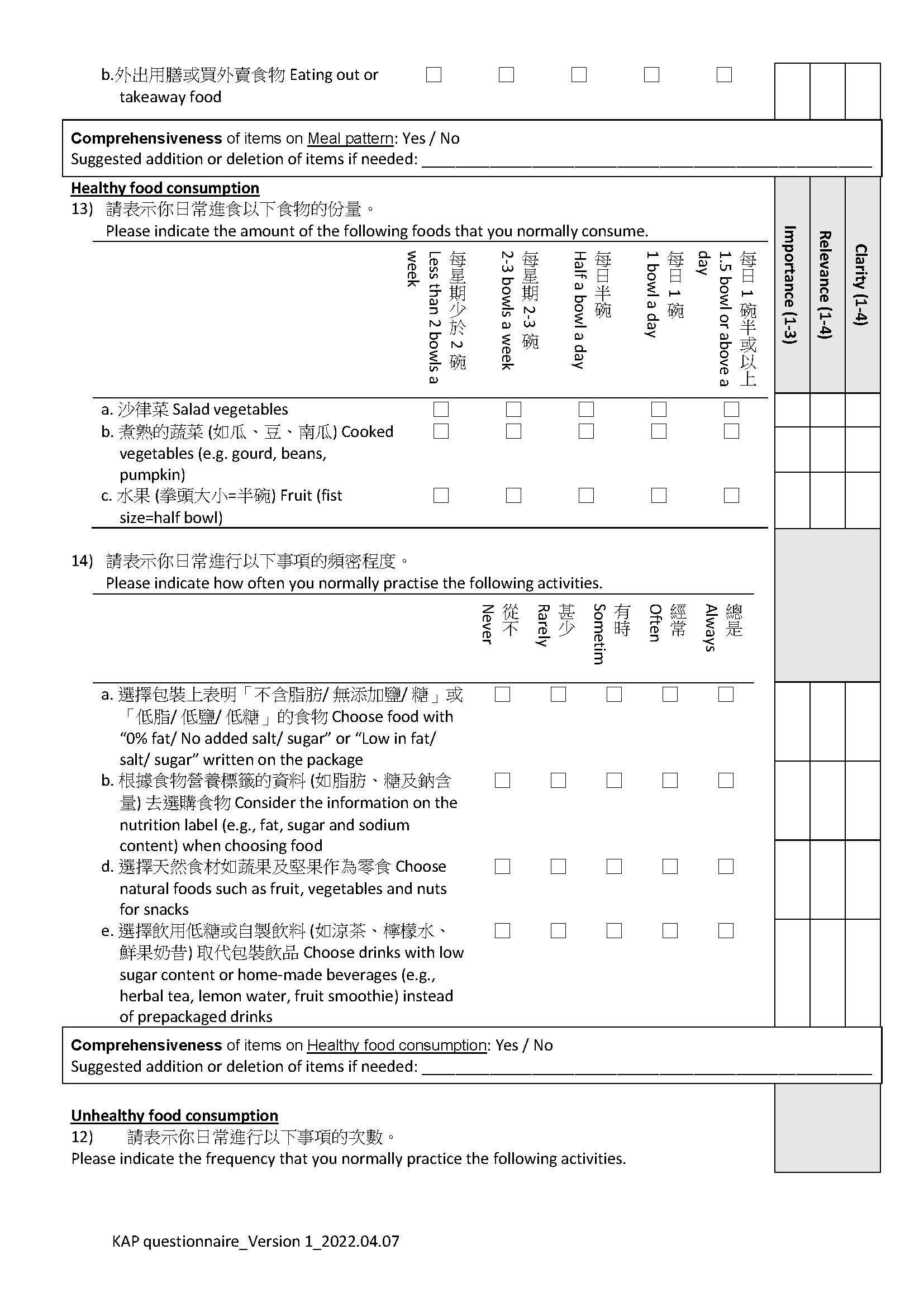

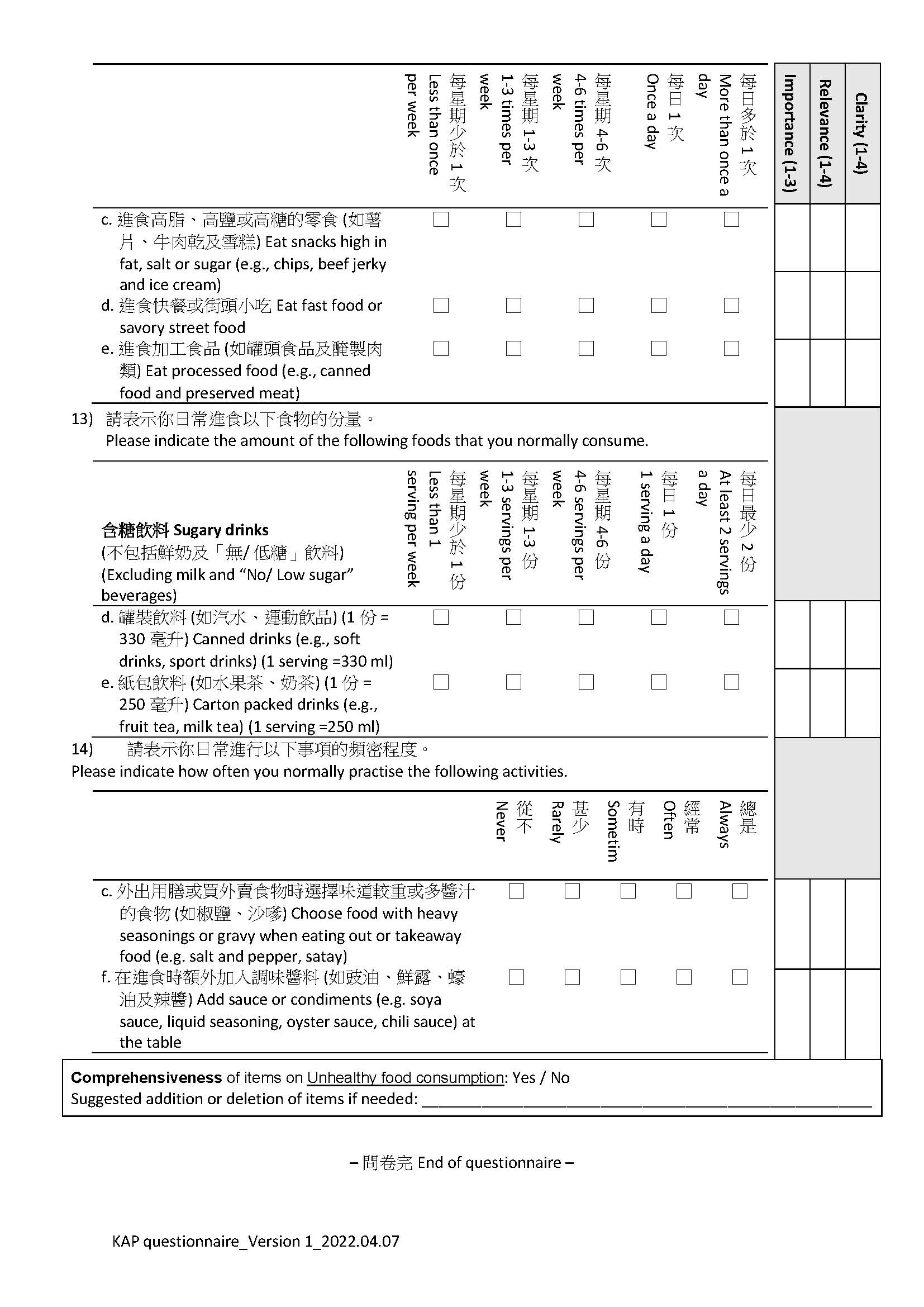

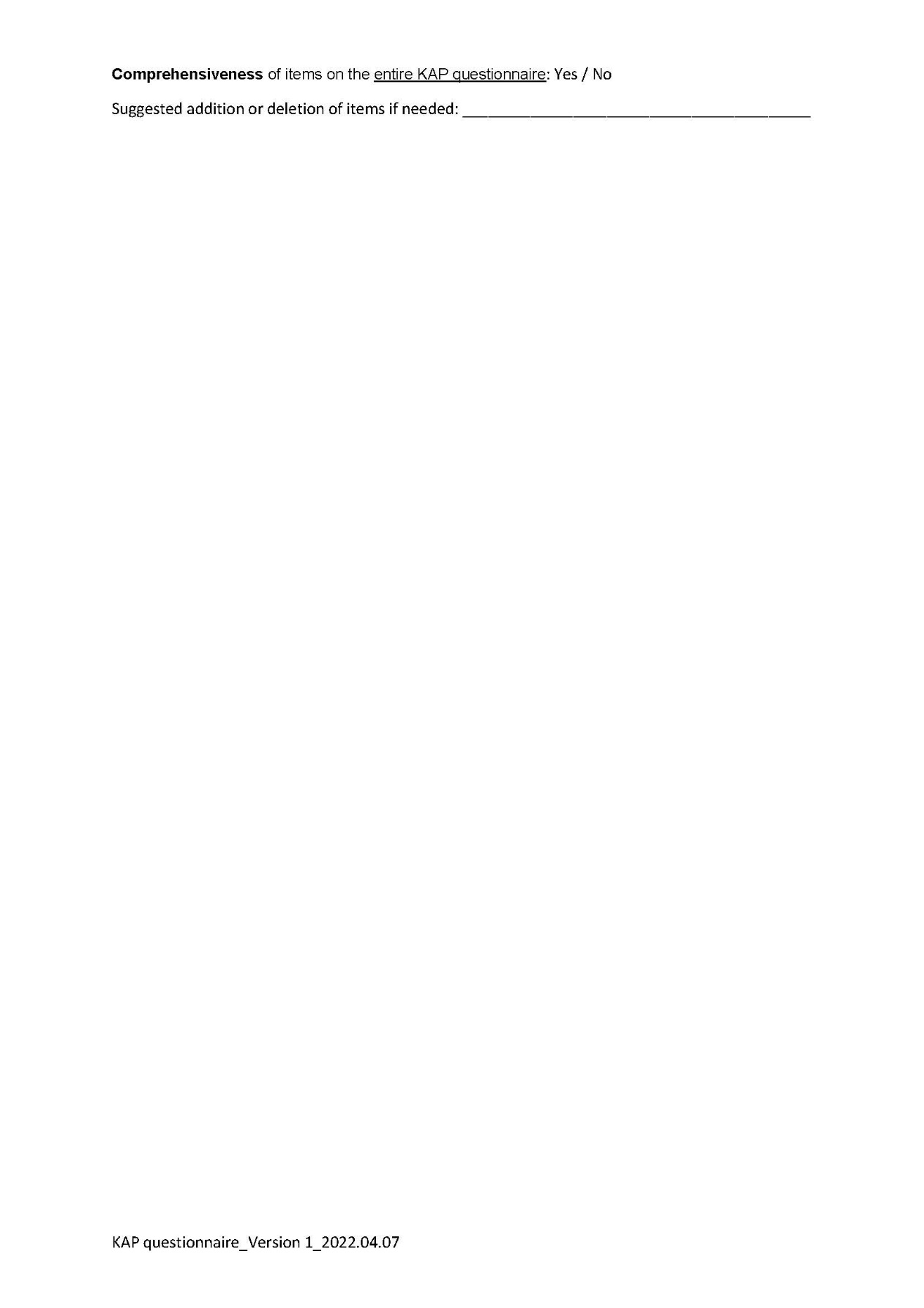

Supplement: Supplementary file 1 [file Data_Sheet_1.DOCX]

**Supplementary Data Sheet 2.** Final draft KAP-HEQ
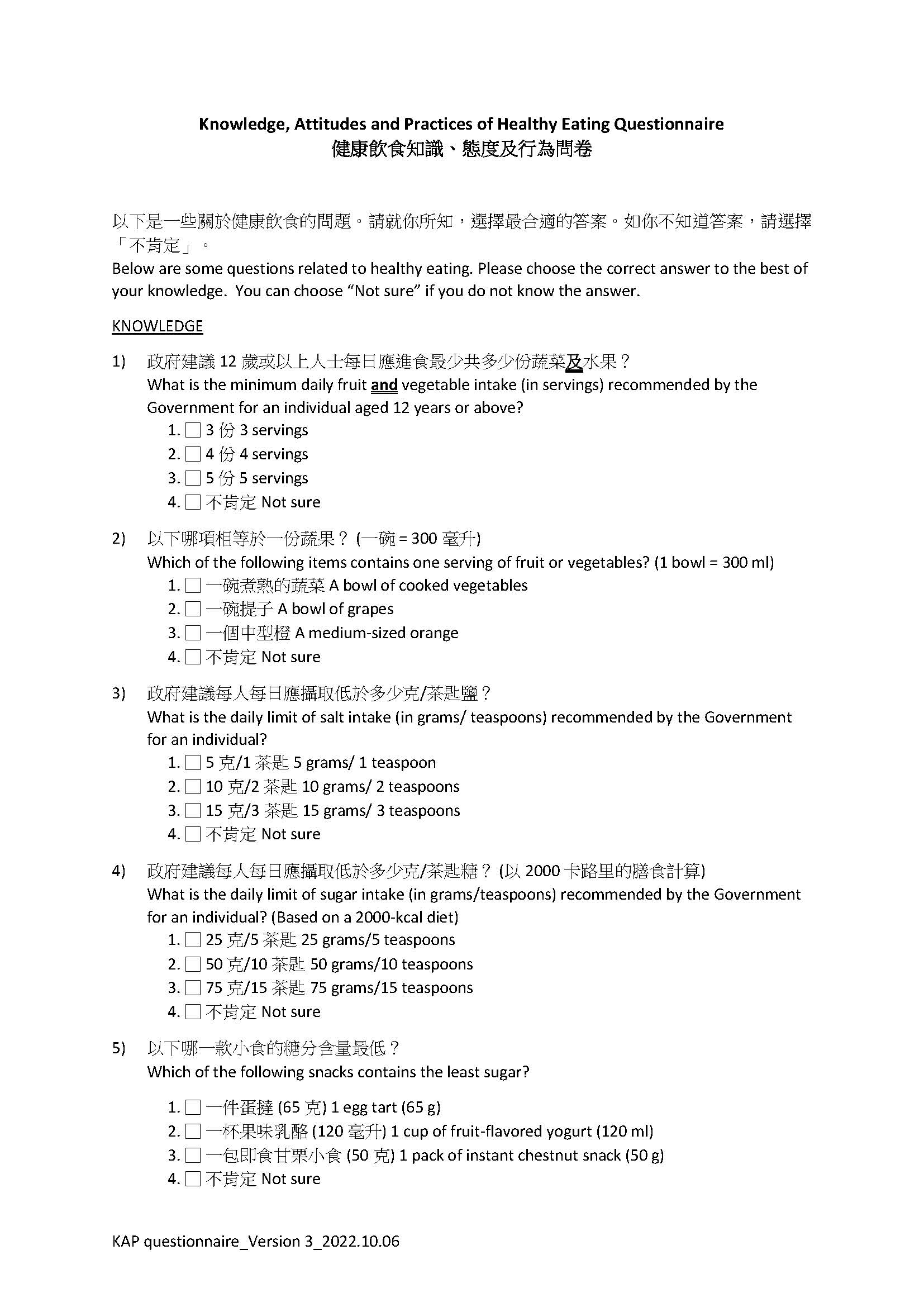


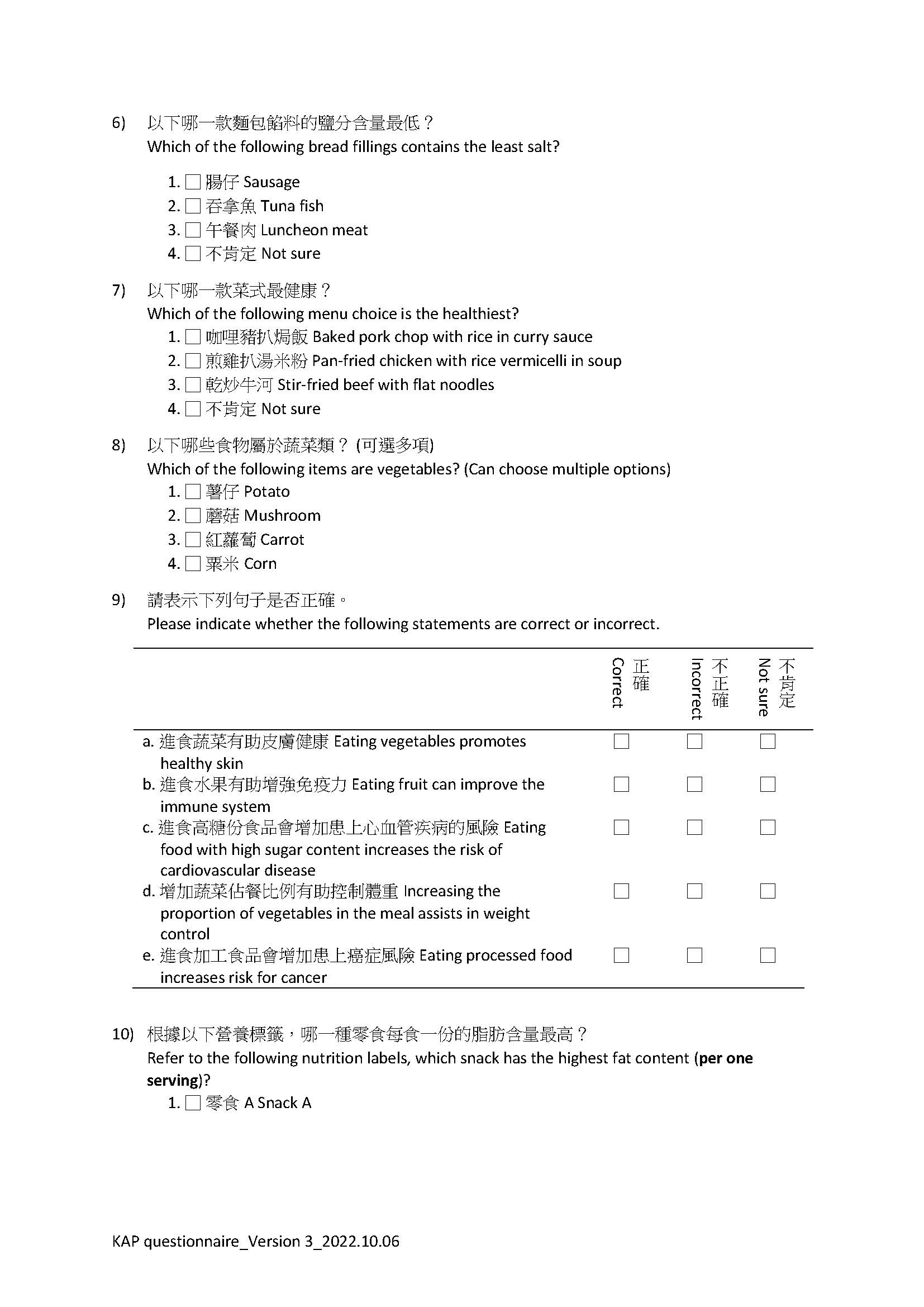

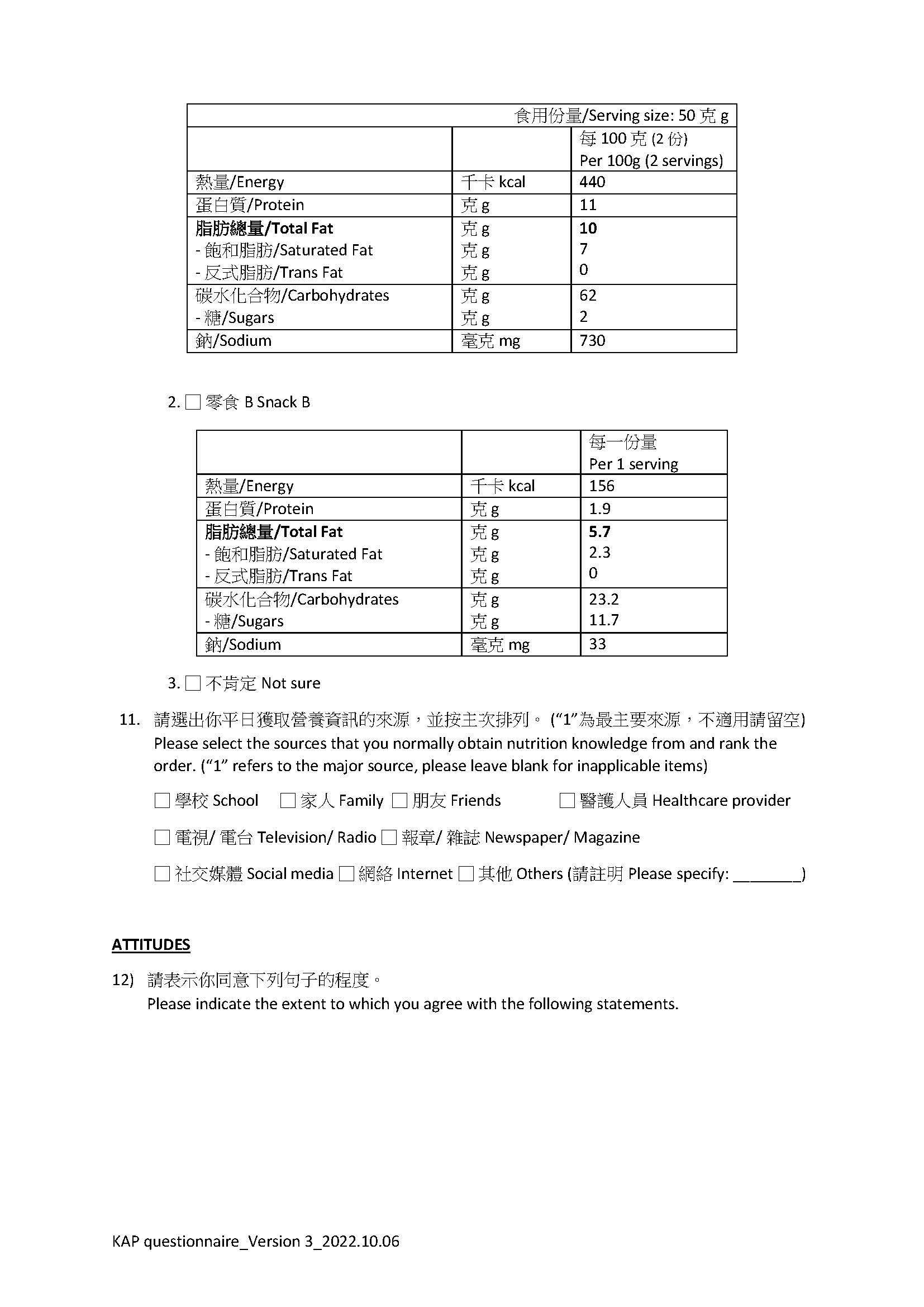

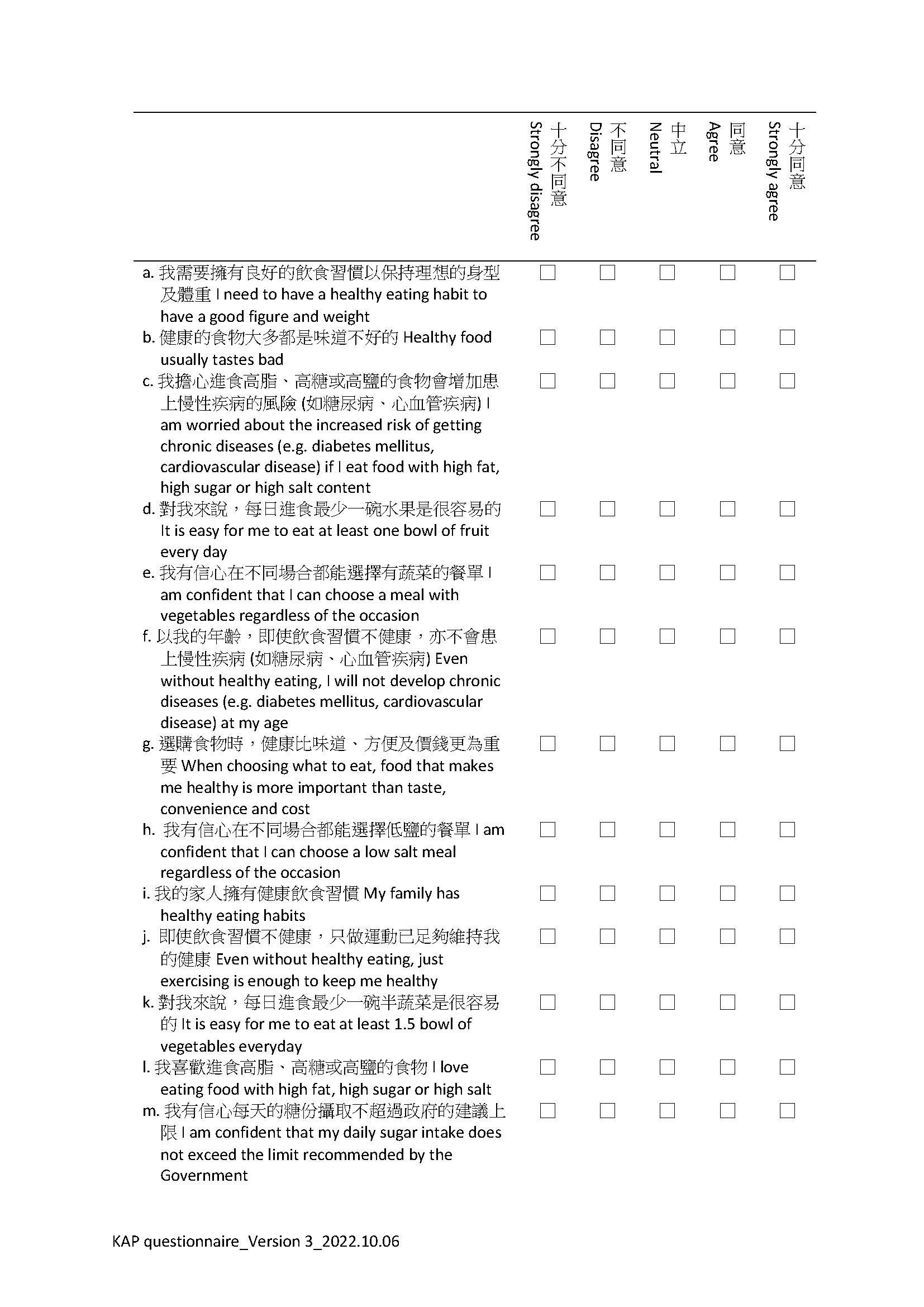

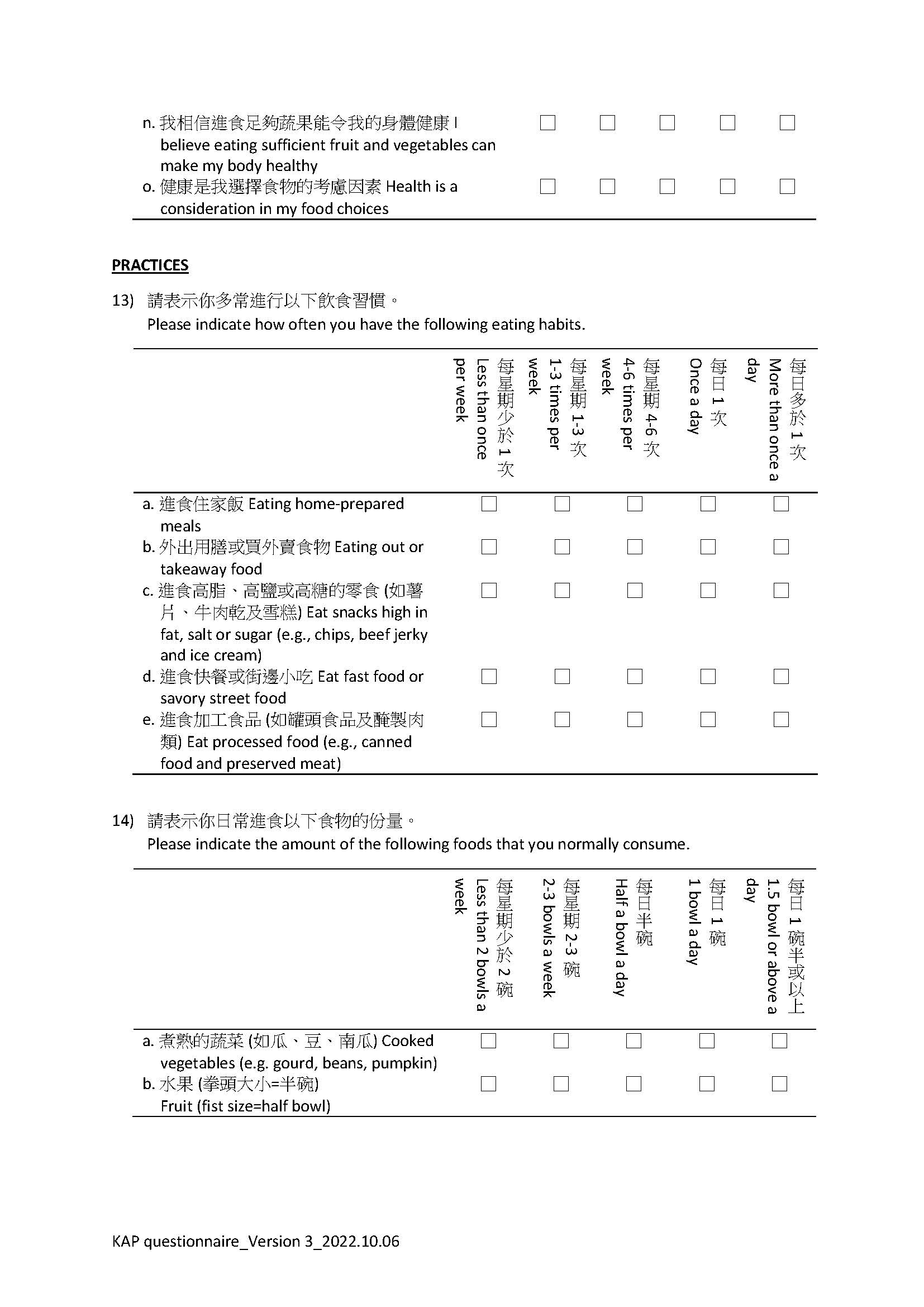

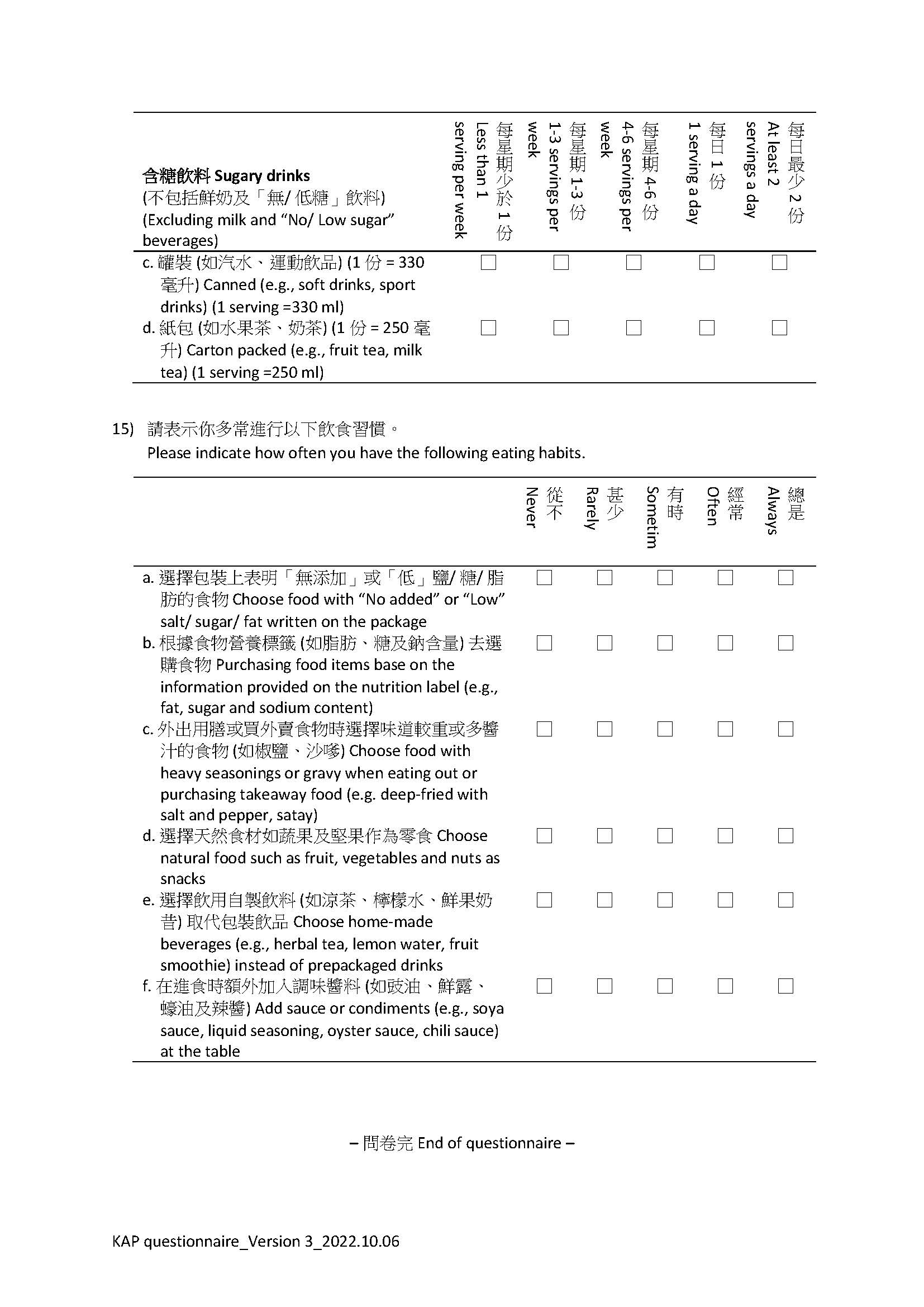

Supplement: Supplementary file 2 [file Data_Sheet_2.DOCX]
