# Supplementary material for: The Knowledge, Attitudes, and Practices of Healthy Eating Questionnaire: a pilot validation study in Chinese families
Source: Front Public Health. 2024 Jul 17;12:1355638. doi: 10.3389/fpubh.2024.1355638 (PMC11288980; doi:10.3389/fpubh.2024.1355638)
Supplement: Supplementary file 3 [file Table_1.DOCX]

**Supplementary Table 1.** Summary of content validity evaluation on KAP-HEQ by expert panel and lay persons

| **Items** | | **Expert review (N=8)** | | | | **Lay person review (N=16)** | | | | | | | |
| --- | --- | --- | --- | --- | --- | --- | --- | --- | --- | --- | --- | --- | --- |
|  |  | CVR | CVI - Relevance | CVI - Clarity | Actions taken | CVR | | CVI - Relevance | | CVI - Clarity | | Actions taken | |
|  |  | (≥ 0.75) | (≥ 0.78) | (≥ 0.78) |  | (≥ 0) | | (≥ 0.75) | | (≥ 0.75) | |  | |
| **Knowledge** | |  |  |  |  |  | |  | |  | |  | |
| *Dietary recommendations* | |  |  |  |  |  | |  | |  | |  | |
| 1 | On FV intake | 0.75 | 1 | **0.75 ^a^** | Reworded | 0.5 | | 0.75 | | 0.88 | |  | |
| 2 | Definition of one serving of FV | 0.75 | 0.88 | 0.88 |  | 0.38 | | 0.88 | | 0.75 | |  | |
| 3 | On salt intake | 0.75 | 0.88 | 1 |  | 0.75 | | 0.81 | | 0.88 | |  | |
| 4 | On sugar intake | 1 | 1 | 1 | Reworded | 0.63 | | 0.81 | | 0.88 | |  | |
| *Food choice* | |  |  |  |  |  | |  | |  | |  | |
| 5 | Healthy food option with low sugar | 0.75 | 1 | 1 |  | **-0.25 ^a^** | | 0.75 | | 0.81 | | Reworded | |
| 6 | Healthy food option with low salt | 0.75 | 1 | 1 |  | **-0.13 ^a^** | | **0.69 ^a^** | | 0.75 | | Reworded | |
| 7 | Healthy eating out option | 0.75 | 0.88 | 1 |  | **-0.25 ^a^** | | 0.81 | | 0.81 | | Reworded | |
| 8 | Examples of vegetables | 0.75 | 1 | 1 |  | 0.38 | | 0.81 | | 0.94 | |  | |
| 9 | Knowledge in reading nutrition label | 1 | 1 | 1 | Reformatted | 0 | | **0.63 ^a^** | | **0.63 ^a^** | | Reformatted | |
| *Health outcomes* | |  |  |  |  |  | |  | |  | |  | |
| 10 | Eating vegetables promotes skin health | **0.5 ^a^** | 0.88 | 0.88 | Retained | 0.13 | | 0.88 | | 1 | |  | |
| 11 | Eating meat is the only way to build muscle mass | 0.75 | 0.88 | 0.88 |  | **-0.38 ^a^** | | **0.56 ^a^** | | **0.69 ^a^** | | Deleted | |
| 12 | Eating fruit can improve the immune system | 1 | 1 | 1 |  | 0.25 | | 0.88 | | 0.94 | |  | |
| 13 | Eating food with high sugar content increases the risk of cardiovascular disease | 1 | 1 | 1 | Reworded | 0.5 | | 0.88 | | 1 | |  | |
| 14 | Increasing the proportion of vegetables in the meal assists in weight control | 1 | 1 | 1 |  | 0.63 | | 0.88 | | 1 | |  | |
| 15 | Eating processed food increases risk for cancer | 1 | 1 | 1 | Reworded | 0.5 | | 0.88 | | 0.94 | |  | |
| **Attitudes** | |  |  |  |  |  | |  | |  | |  | |
| *Outcome expectation* | |  |  |  |  |  | |  | |  | |  | |
| 16 | Avoid unhealthy eating habits to maintain health | **0.5 ^a^** | 0.88 | 0.88 | Deleted | 0.5 | | 0.88 | | 1 | |  | |
| 17 | Healthy eating habit for a good figure and weight | 0.75 | 1 | 1 | Reworded | 0.13 | | 0.88 | | 1 | |  | |
| 18 | Risk of getting chronic diseases with unhealthy eating | 0.75 | 1 | 1 |  | 0.25 | | 0.94 | | 0.94 | |  | |
| 19 | Risk of chronic disease at my age | 0.75 | 1 | 1 |  | 0.13 | | **0.69 ^a^** | | 0.81 | | Reworded | |
| 20 | Exercising is enough to keep me healthy | 1 | 1 | 1 | Reworded | **-0.38 ^a^** | | 0.81 | | 0.81 | | Retained | |
| 21 | Reduced risk of getting diseases with FV intake | 0.75 | 1 | 1 |  | **-0.13 ^a^** | | 0.81 | | 0.88 | | Reworded | |
| *Preference & subjective norm* | |  |  |  |  |  | |  | |  | |  | |
| 22 | Affective feeling towards healthy food | 0.75 | 1 | 1 | Reworded | **-0.38 ^a^** | | 0.75 | | 0.94 | | Retained | |
| 23 | Subjective norm among friends | 0.75 | 0.88 | 1 |  | **-0.38 ^a^** | | 0.88 | | 0.94 | | Deleted | |
| 24 | Priority of health among other motives | 1 | 1 | 1 |  | 0 | | 0.75 | | 0.94 | |  | |
| 25 | Subjective norm among family | 1 | 0.88 | 1 |  | 0 | | 0.81 | | 0.94 | |  | |
| 26 | Affective feeling towards unhealthy food | 1 | 1 | 1 |  | 0 | | 0.88 | | 0.88 | |  | |
| 27 | Consideration of health in food choice | 1 | 1 | 1 |  | **-0.13 ^a^** | | 0.94 | | 1 | | Reworded | |
| *Self-efficacy* | |  |  |  |  |  | |  | |  | |  | |
| 28 | Perceived ability to eat sufficient fruit | 1 | 1 | 1 | Reworded | **-0.13 ^a^** | | 0.75 | | 0.81 | | Reworded | |
| 29 | Self-efficacy on vegetable intake | 0.75 | 0.88 | 0.88 |  | **-0.13 ^a^** | | 0.88 | | 0.88 | | Reworded | |
| 30 | Self-efficacy on low salt intake | 0.75 | 1 | 1 |  | **-0.13 ^a^** | | 0.81 | | 0.81 | | Reworded | |
| 31 | Perceived ability to eat sufficient vegetables | 1 | 1 | 0.88 | Reworded | 0 | | 0.75 | | 0.75 | | Reworded | |
| 32 | Self-efficacy on low sugar intake | 0.75 | 0.88 | **0.75 ^a^** | Reworded | **-0.13 ^a^** | | 0.81 | | 0.81 | | Reworded | |
| **Practices** | |  |  |  |  |  | |  | |  | |  | |
| *Meal pattern* | |  |  |  |  |  | |  | |  | |  | |
| 33 | Frequency of eating home-prepared meals | 0.75 | 0.88 | 1 |  | 0.5 | | 0.81 | | 1 | |  | |
| 34 | Frequency of eating out or takeaway food | 1 | 1 | 1 |  | 0.38 | | 0.75 | | 0.88 | |  | |
| *Healthy food consumption* | |  |  |  |  |  | |  | |  | |  | |
| 35 | Quantity of salad vegetable intake | 0.75 | 1 | 1 |  | **-0.5 ^a^** | | 0.75 | | 0.88 | | Deleted | |
| 36 | Quantity of cooked vegetable intake | 0.75 | 1 | 1 |  | 0.25 | | 0.94 | | 0.81 | |  | |
| 37 | Quantity of fruit intake | 1 | 1 | 1 |  | 0.5 | | 0.94 | | 0.94 | |  | |
| 38 | Food decision by nutrition claim on package | 1 | 1 | 0.88 | Reworded | 0.13 | | 0.94 | | 0.88 | |  | |
| 39 | Food decision by nutrition label | 1 | 1 | 1 |  | 0.13 | | 0.88 | | 0.75 | |  | |
| 40 | Choosing healthy options as snacks | 1 | 1 | 1 |  | 0.25 | | 0.88 | | 0.88 | |  | |
| 41 | Healthy alternatives to prepackaged drinks | 1 | 0.88 | **0.75 ^a^** | Reworded | 0.13 | | 0.88 | | 0.94 | |  | |
| *Unhealthy food consumption* | |  |  |  |  |  | |  | |  | |  | |
| 42 | Frequency of eating unhealthy snacks | 1 | 1 | 1 |  | 0.38 | | 0.94 | | 0.94 | |  | |
| 43 | Frequency of eating fast food or street savory snacks | 1 | 0.88 | 0.88 |  | 0.13 | | 0.81 | | 0.88 | |  | |
| 44 | Frequency of eating processed food, e.g. instant noodles, luncheon meat | 1 | 1 | 1 |  | 0.38 | 0.88 | | 0.88 | |  | |  |
| 45 | Frequency of drinking canned SSB | 1 | 1 | 1 |  | **-0.13 ^a^** | 0.81 | | 0.81 | | Retained | |  |
| 46 | Frequency of drinking carton packed SSB | 1 | 1 | 1 |  | **-0.13 ^a^** | 0.81 | | 0.81 | | Retained | |  |
| 47 | Choosing unhealthy meals when eating out | 1 | 1 | 1 |  | 0 | 1 | | 1 | |  | |  |
| 48 | Adding seasonings at the table | 0.75 | 1 | 1 |  | 0 | 1 | | 1 | |  | |  |
| CVI – content validity index, CVR – content validity ratio, FV – fruit and vegetables, SSB – sugar-sweetened beverages Note: ^a^ Values below the standard. | | | | | | | | | | | | |  |
