# Supplementary material for: The Knowledge, Attitudes, and Practices of Healthy Eating Questionnaire: a pilot validation study in Chinese families
Source: Front Public Health. 2024 Jul 17;12:1355638. doi: 10.3389/fpubh.2024.1355638 (PMC11288980; doi:10.3389/fpubh.2024.1355638)
Supplement: Supplementary file 4 [file Table_2.DOCX]

**Supplementary Table 2.** Item-scale correlations of KAP scales among adolescents (N=60) and parents (N=60)

| **Item** | **Adolescents** | | | **Parents** | | |  |  |  |  |
| --- | --- | --- | --- | --- | --- | --- | --- | --- | --- | --- |
|  | **Scale** | | | **Scale** | | |  |  |  |  |
|  | **Knowledge** | **Attitudes** | **Practices** | **Knowledge** | **Attitudes** | **Practices** | |  |  |  |
| **Knowledge** | | | | | | |  |  |  |  |
| Q1 | **0.24** | 0.10 | 0.09 | **-0.08** | 0.15 | 0.26* | |  |  |  |
| Q2 | **-0.05** | 0.00 | 0.01 | **0.43***** | 0.11 | 0.09 | |  |  |  |
| Q3 | **-0.05** | -0.04 | 0.21 | **-0.08** | 0.19 | 0.14 | |  |  |  |
| Q4 | **0.22** | -0.07 | 0.13 | **0.16** | 0.23 | 0.21 | |  |  |  |
| Q5 | **0.16** | 0.01 | 0.02 | **0.5***** | 0.27* | 0.15 | |  |  |  |
| Q6 | **0.18** | -0.12 | -0.07 | **0.21** | 0.14 | 0.19 | |  |  |  |
| Q7 | **0.08** | -0.24 | -0.09 | **0.28*** | 0.24 | -0.05 | |  |  |  |
| Q8 | **0.00** | 0.21 | -0.01 | **0.29*** | 0.04 | 0.00 | |  |  |  |
| Q9a | **0.46***** | -0.02 | 0.14 | **0.00** | 0.26* | 0.22 | |  |  |  |
| Q9b | **0.34**** | -0.06 | 0.13 | **0.33*** | 0.25 | 0.13 | |  |  |  |
| Q9c | **0.34**** | 0.04 | 0.16 | **0.29*** | 0.08 | 0.03 | |  |  |  |
| Q9d | **0.40**** | -0.04 | 0.00 | **0.25** | 0.11 | 0.07 | |  |  |  |
| Q9e | **0.30*** | -0.05 | 0.14 | **0.27*** | 0.14 | 0.03 | |  |  |  |
| Q10 | **0.18** | -0.08 | -0.08 | **0.02** | -0.02 | 0.12 | |  |  |  |
| **Attitudes** | | | | | | |  | |  |  |
| Q12a | 0.07 | **0.63***** | 0.18 | 0.23 | **0.5***** | 0.16 | |  |  |  |
| Q12b | -0.04 | **-0.04** | 0.34** | -0.01 | **-0.10** | 0.17 | |  |  |  |
| Q12c | 0.02 | **0.65***** | 0.02 | 0.23 | **0.22** | 0.02 | |  |  |  |
| Q12d | 0.19 | **0.46***** | 0.21 | 0.39** | **0.66***** | 0.37** | |  |  |  |
| Q12e | -0.12 | **0.36**** | 0.13 | 0.17 | **0.75***** | 0.44*** | |  |  |  |
| Q12f | 0.07 | **0.23** | 0.18 | 0.19 | **0.30*** | 0.21 | |  |  |  |
| Q12g | -0.08 | **0.45***** | 0.02 | 0.23 | **0.64***** | 0.51*** | |  |  |  |
| Q12h | -0.18 | **0.28*** | 0.1 | 0.19 | **0.66***** | 0.40** | |  |  |  |
| Q12i | 0.03 | **0.53***** | 0.22 | 0.03 | **0.59***** | 0.47*** | |  |  |  |
| Q12j | 0.01 | **0.20** | 0.14 | 0.28* | **0.12** | 0.22 | |  |  |  |
| Q12k | -0.08 | **0.47***** | 0.29* | 0.33* | **0.69***** | 0.54*** | |  |  |  |
| Q12l | 0.07 | **0.34**** | 0.38** | 0.22 | **0.57***** | 0.72*** | |  |  |  |
| Q12m | -0.15 | **0.30*** | 0.18 | 0.25 | **0.65***** | 0.66*** | |  |  |  |
| Q12n | 0.06 | **0.54***** | -0.08 | 0.18 | **0.58***** | 0.30* | |  |  |  |
| Q12o | -0.09 | **0.57***** | 0.07 | 0.22 | **0.77***** | 0.51*** | |  |  |  |
| **Practices** | | | | | | |  | |  |  |
| Q13a | 0.10 | 0.15 | **0.23** | 0.24 | 0.44*** | **0.33*** | |  |  |  |
| Q13b | 0.04 | 0.21 | **0.41**** | -0.01 | 0.20 | **0.43***** | |  |  |  |
| Q13c | -0.02 | 0.13 | **0.59***** | 0.02 | 0.43*** | **0.50***** | |  |  |  |
| Q13d | 0.24 | 0.19 | **0.57***** | 0.11 | 0.40** | **0.60***** | |  |  |  |
| Q13e | 0.06 | 0.10 | **0.44***** | 0.04 | 0.13 | **0.26*** | |  |  |  |
| Q14a | 0.00 | 0.21 | **0.42***** | 0.13 | 0.33** | **0.42***** | |  |  |  |
| Q14b | 0.05 | 0.18 | **0.29*** | 0.20 | 0.38** | **0.46***** | |  |  |  |
| Q14c | 0.24 | 0.11 | **0.52***** | 0.19 | 0.27* | **0.35**** | |  |  |  |
| Q14d | 0.10 | 0.16 | **0.60***** | 0.09 | 0.39** | **0.51***** | |  |  |  |
| Q15a | 0.05 | 0.21 | **0.22** | 0.20 | 0.40** | **0.26*** | |  |  |  |
| Q15b | 0.18 | 0.05 | **0.29*** | 0.12 | 0.42*** | **0.43***** | |  |  |  |
| Q15c | 0.27* | 0.22 | **0.26*** | -0.01 | 0.37** | **0.34**** | |  |  |  |
| Q15d | 0.02 | 0.04 | **0.4**** | 0.28* | 0.44*** | **0.64***** | |  |  |  |
| Q15e | -0.06 | 0.11 | **0.18** | 0.14 | 0.34** | **0.50***** | |  |  |  |
| Q15f | 0.11 | 0.10 | **0.28*** | 0.00 | 0.23 | **0.41**** | |  |  |  |
| Notes: All coefficients in bold were corrected for overlap by exclusion of the item score from the scale score calculation * p-value < 0.05; ** p-value < 0.01; *** p-value < 0.001 by Spearman Correlation Test | | | | | | |  |  |  |  |

**Supplementary Table 3.** Internal consistency and inter-scale correlations of KAP scales among adolescents (N=60) and parents (N=60)

| **Scale** | **Cronbach's alpha** | **Inter-scale correlation** | |
| --- | --- | --- | --- |
|  |  | **Attitudes** | **Practices** |
| **Adolescents** |  |  |  |
| **Overall** | 0.77 |  |  |
| **Knowledge** | 0.56 | 0.00 | 0.16 |
| **Attitudes** | 0.76 |  | 0.33** |
| **Practices** | 0.77 |  |  |
| **Parents** |  |  |  |
| **Overall** | 0.89 |  |  |
| **Knowledge** | 0.52 | 0.38** | 0.23 |
| **Attitudes** | 0.87 |  | 0.62*** |
| **Practices** | 0.79 |  |  |
| Notes: ** p-value < 0.01; *** p-value < 0.001 by Spearman Correlation Test | | | |

**Supplementary Table 4.** Test-retest reliability of KAP scales among adolescents (N=30) and parents (N=30)

| **Scale** | **ICC** | **Mean (T0)** | **Mean (T1)** | **P-value of paired t-test** | **Cohen's d effect size** |
| --- | --- | --- | --- | --- | --- |
| **Adolescents** | | | | | |
| **Overall** | 0.77 | 59.21 | 60.40 | 0.40 | -0.16 |
| **Knowledge** | 0.43 | 62.29 | 63.75 | 0.71 | -0.07 |
| **Attitudes** | 0.77 | 56.11 | 57.39 | 0.45 | -0.14 |
| **Practices** | 0.60 | 59.22 | 60.06 | 0.68 | -0.08 |
| **Parents** | | | | | |
| **Overall** | 0.89 | 66.21 | 66.89 | 0.62 | -0.09 |
| **Knowledge** | 0.78 | 63.75 | 66.67 | 0.19 | -0.24 |
| **Attitudes** | 0.85 | 64.89 | 65.11 | 0.92 | -0.02 |
| **Practices** | 0.91 | 70.00 | 68.89 | 0.43 | 0.15 |
| ICC – intraclass correlation coefficient, T0 – first test, T1 – second test in 2 weeks | | | | | |

**Supplementary Table 5.** Known-group comparison of KAP scales among adolescents (N=60) and parents (N=60)

| **Scale** | **Adolescents** | | | **Parents** | | |
| --- | --- | --- | --- | --- | --- | --- |
|  | **Mean difference** | **P-value** | **Cohen's d effect size** | **Mean difference** | **P-value** | **Cohen's d effect size** |
|  | **FV intake** (≥ 4 servings vs < 4 servings daily) | | | | | |
|  | (n=13 vs n=47) | | | (n=23 vs n=37) | | |
| **Overall ^a^** | 4.39* | 0.02 | 0.56 | 9.6** | 0.00 | 0.99 |
| **Knowledge** | 2.43 | 0.50 | 0.15 | 8.47* | 0.03 | 0.60 |
| **Attitudes** | 1.72 | 0.65 | 0.16 | 10.96** | 0.01 | 0.81 |
| **Practices ^a^** | 9.02** | 0.00 | 0.83 | 9.37** | 0.01 | 0.78 |
|  | **Parental education** (≥ 13 years vs < 13 years) | | | | | |
|  | (n=30 vs n=30) | | | (n=30 vs n=30) | | |
| **Overall** | 0.13 | 0.95 | 0.02 | 8.15** | 0.00 | 0.81 |
| **Knowledge** | 5.00 | 0.22 | 0.32 | 6.46 | 0.09 | 0.45 |
| **Attitudes** | -0.89 | 0.76 | -0.08 | 10.83** | 0.00 | 0.80 |
| **Practices** | -3.72 | 0.20 | -0.34 | 7.17* | 0.03 | 0.57 |
|  | **Household income** (≥ $20,000 vs < $20,000) | | | | | |
|  | (n=23 vs n=37) | | | (n=23 vs n=37) | | |
| **Overall** | -0.31 | 0.88 | -0.04 | 7.06* | 0.02 | 0.68 |
| **Knowledge** | 3.80 | 0.40 | 0.24 | 14.2*** | <.001 | 1.09 |
| **Attitudes** | -6.4* | 0.03 | -0.60 | 3.21 | 0.44 | 0.22 |
| **Practices** | 1.67 | 0.57 | 0.15 | 3.78 | 0.32 | 0.29 |
| Notes: ^a^ Corrected for overlap by exclusion of the item score of FV intake from the scale score calculation  * p-value < 0.05; ** p-value < 0.01; *** p-value < 0.001 by Spearman Correlation Test | | | | | | |

**Supplementary Table 6.** Age and KAP scale scores among female (N=33) and male (N=27) adolescents

|  | **Female** | | **Male** | | **P-value** | | |  |
| --- | --- | --- | --- | --- | --- | --- | --- | --- |
|  | n/ Mean | %/ SD | n/ Mean | %/ SD |  | | |  |
| **Age** | 14.79 | ±1.69 | 16.26 | ±1.75 | 0.00** | | |  |
| 12-13 | 8 | 24.24% | 2 | 7.41% | 0.03* | | |  |
| 14-15 | 14 | 42.42% | 8 | 29.63% |  |  |  |  |
| 16-17 | 9 | 27.27% | 8 | 29.63% |  |  |  |  |
| 18-19 | 2 | 6.06% | 9 | 33.33% |  |  |  |  |
| **KAP of Healthy Eating score** | |  |  |  |  | | |  |
| Overall | 58.41 | ±7.64 | 56.23 | ±8.18 | 0.30 | | |  |
| Knowledge | 57.95 | ±14.00 | 57.41 | ±17.93 | 0.90 | | |  |
| Attitudes | 55.81 | ±11.33 | 55.86 | ±10.80 | 0.98 | | |  |
| Practices | 61.46 | ±10.54 | 55.43 | ±11.01 | 0.04* | | |  |
| Notes: * p-value < 0.05; ** p-value < 0.01 by Spearman Correlation Test | | | | | |  |  | |

**Supplementary Table 7.** Item-scale correlations of KAP scales among female (N=33) and male (N=27) adolescents

| **Item** | **Female adolescents** | | | **Male adolescents** | | |  |  |  |  |
| --- | --- | --- | --- | --- | --- | --- | --- | --- | --- | --- |
|  | **Scale** | | | **Scale** | | |  |  |  |  |
|  | **Knowledge** | **Attitudes** | **Practices** | **Knowledge** | **Attitudes** | **Practices** | |  |  |  |
| **Knowledge** | | | | | | |  |  |  |  |
| Q1 | **0.11** | 0.22 | 0.18 | **0.41*** | 0.10 | 0.09 | |  |  |  |
| Q2 | **0.15** | 0.17 | 0.50** | **-0.18** | 0.00 | 0.01 | |  |  |  |
| Q3 | **-0.07** | -0.26 | 0.14 | **-0.03** | -0.04 | 0.21 | |  |  |  |
| Q4 | **0.35*** | -0.15 | 0.11 | **0.10** | -0.07 | 0.13 | |  |  |  |
| Q5 | **0.12** | 0.12 | 0.03 | **0.21** | 0.01 | 0.02 | |  |  |  |
| Q6 | **0.11** | -0.09 | -0.01 | **0.35** | -0.12 | -0.07 | |  |  |  |
| Q7 | **-0.04** | -0.24 | -0.05 | **0.23** | -0.24 | -0.09 | |  |  |  |
| Q8 | **-0.19** | 0.23 | 0.10 | **0.23** | 0.21 | -0.01 | |  |  |  |
| Q9a | **0.34** | -0.07 | 0.20 | **0.59**** | -0.02 | 0.14 | |  |  |  |
| Q9b | **0.42*** | -0.09 | 0.36* | **0.30** | -0.06 | 0.13 | |  |  |  |
| Q9c | **0.31** | 0.01 | 0.39* | **0.38*** | 0.04 | 0.16 | |  |  |  |
| Q9d | **0.33** | -0.09 | 0.10 | **0.47*** | -0.04 | 0.00 | |  |  |  |
| Q9e | **0.31** | -0.21 | 0.12 | **0.33** | -0.05 | 0.14 | |  |  |  |
| Q10 | **0.10** | 0.06 | 0.05 | **0.30** | -0.08 | -0.08 | |  |  |  |
| **Attitudes** | | | | | | |  | |  |  |
| Q12a | -0.03 | **0.71***** | 0.15 | 0.17 | **0.54**** | 0.28 | |  |  |  |
| Q12b | -0.04 | **-0.28** | 0.24 | -0.07 | **0.24** | 0.36 | |  |  |  |
| Q12c | -0.11 | **0.63***** | -0.15 | 0.18 | **0.70***** | 0.23 | |  |  |  |
| Q12d | 0.17 | **0.38*** | 0.03 | 0.20 | **0.59**** | 0.45* | |  |  |  |
| Q12e | -0.16 | **0.38*** | 0.09 | -0.07 | **0.38*** | 0.32 | |  |  |  |
| Q12f | 0.15 | **-0.02** | 0.14 | -0.04 | **0.60**** | 0.22 | |  |  |  |
| Q12g | -0.35* | **0.53**** | 0.02 | 0.22 | **0.37** | 0.02 | |  |  |  |
| Q12h | -0.18 | **0.19** | -0.07 | -0.20 | **0.44*** | 0.41* | |  |  |  |
| Q12i | 0.02 | **0.47**** | 0.04 | 0.03 | **0.61***** | 0.40* | |  |  |  |
| Q12j | 0.01 | **0.01** | 0.14 | 0.00 | **0.45*** | 0.16 | |  |  |  |
| Q12k | -0.15 | **0.47**** | 0.15 | -0.01 | **0.51**** | 0.48* | |  |  |  |
| Q12l | 0.16 | **0.25** | 0.39* | -0.04 | **0.48*** | 0.28 | |  |  |  |
| Q12m | -0.03 | **0.26** | 0.12 | -0.30 | **0.39*** | 0.24 | |  |  |  |
| Q12n | 0.01 | **0.71***** | -0.04 | 0.12 | **0.32** | -0.15 | |  |  |  |
| Q12o | -0.15 | **0.69***** | -0.05 | -0.04 | **0.56**** | 0.27 | |  |  |  |
| **Practices** | | | | | | |  | |  |  |
| Q13a | 0.15 | 0.23 | **0.28** | 0.08 | 0.04 | **0.11** | |  |  |  |
| Q13b | 0.13 | 0.22 | **0.28** | -0.06 | 0.24 | **0.48*** | |  |  |  |
| Q13c | 0.15 | 0.07 | **0.55***** | -0.21 | 0.23 | **0.57**** | |  |  |  |
| Q13d | 0.28 | -0.05 | **0.42*** | 0.22 | 0.37 | **0.65***** | |  |  |  |
| Q13e | 0.31 | 0.04 | **0.41*** | -0.17 | 0.16 | **0.42*** | |  |  |  |
| Q14a | 0.00 | 0.12 | **0.41*** | 0.00 | 0.35 | **0.39*** | |  |  |  |
| Q14b | 0.05 | 0.15 | **0.03** | 0.00 | 0.21 | **0.52**** | |  |  |  |
| Q14c | 0.45** | 0.04 | **0.52**** | -0.05 | 0.23 | **0.46*** | |  |  |  |
| Q14d | 0.20 | 0.10 | **0.71***** | -0.02 | 0.24 | **0.53**** | |  |  |  |
| Q15a | 0.05 | 0.09 | **0.17** | 0.05 | 0.36 | **0.24** | |  |  |  |
| Q15b | 0.23 | -0.08 | **0.31** | 0.17 | 0.25 | **0.34** | |  |  |  |
| Q15c | 0.17 | 0.31 | **0.34** | 0.38 | 0.08 | **0.10** | |  |  |  |
| Q15d | 0.12 | 0.03 | **0.32** | -0.08 | 0.10 | **0.42*** | |  |  |  |
| Q15e | 0.05 | 0.01 | **0.01** | -0.23 | 0.26 | **0.44*** | |  |  |  |
| Q15f | 0.14 | -0.18 | **0.53**** | 0.08 | 0.40* | **0.32** | |  |  |  |
| Notes: All coefficients in bold were corrected for overlap by exclusion of the item score from the scale score calculation * p-value < 0.05; ** p-value < 0.01; *** p-value < 0.001 by Spearman Correlation Test | | | | | | |  |  |  |  |

**Supplementary Table 8.** Internal consistency and inter-scale correlations of KAP scales among female (N=33) and male (N=27) adolescents

| **Scale** | **Cronbach's alpha** | **Inter-scale correlation** | |
| --- | --- | --- | --- |
|  |  | **Attitudes** | **Practices** |
| **Female adolescents** | |  |  |
| **Overall** | 0.71 |  |  |
| **Knowledge** | 0.45 | 0.00 | 0.30 |
| **Attitudes** | 0.72 |  | 0.22 |
| **Practices** | 0.73 |  |  |
| **Male adolescents** |  |  |  |
| **Overall** | 0.83 |  |  |
| **Knowledge** | 0.67 | 0.03 | 0.00 |
| **Attitudes** | 0.83 |  | 0.47* |
| **Practices** | 0.79 |  |  |
| Notes: * p-value < 0.05 by Spearman Correlation Test | | | |
